# Three-dimensional vascular microenvironment landscape in human glioblastoma

**DOI:** 10.1186/s40478-020-01115-0

**Published:** 2021-02-12

**Authors:** George P. Cribaro, Elena Saavedra-López, Leire Romarate, Izaskun Mitxitorena, Laura R. Díaz, Paola V. Casanova, Meritxell Roig-Martínez, José M. Gallego, Ana Perez-Vallés, Carlos Barcia

**Affiliations:** 1 Department of Biochemistry and Molecular Biology, Institut de Neurociències, School of Medicine, Lab M2-107, Office M2-117, Universitat Autònoma de Barcelona, 08193 Bellaterra, Barcelona, Spain; 2 Department of Neurosurgery & Department of Pathology, Valencia General Hospital, Valencia, Spain

**Keywords:** Glioblastoma, Blood vessels, Microglia, Macrophages, Astrocytes, T cells, Endothelium, Basement membrane, Tumor microenvironments

## Abstract

The cellular complexity of glioblastoma microenvironments is still poorly understood. In-depth, cell-resolution tissue analyses of human material are rare but highly necessary to understand the biology of this deadly tumor. Here we present a unique 3D visualization revealing the cellular composition of human GBM in detail and considering its critical association with the neo-vascular niche. Our images show a complex vascular map of human 3D biopsies with increased vascular heterogeneity and altered spatial relationship with astrocytes or glioma-cell counterparts. High-resolution analysis of the structural layers of the blood brain barrier showed a multilayered fenestration of endothelium and basement membrane. Careful examination of T cell position and migration relative to vascular walls revealed increased infiltration corresponding with tumor proliferation. In addition, the analysis of the myeloid landscape not only showed a volumetric increase in glioma-associated microglia and macrophages relative to GBM proliferation but also revealed distinct phenotypes in tumor nest and stroma. Images and data sets are available on demand as a resource for public access.

## Introduction

Despite the recent progress of successful immunotherapeutic options for many types of cancers, glioblastoma (GBM) remains incurable [9, 16]. The aggressive nature of this brain tumor causes a rapid and severe spread of malignant cells in brain tissue with irreparable loss. The extremely complex cellular structure of GBM that enables this invasiveness is still poorly understood and deserves a detailed microanatomical study to comprehend its biology.

Importantly, clinical histopathological examinations are generally based on thin, paraffin-embedded or cryopreserved sections with simultaneous staining of one or two markers, only allowing a two-dimensional visualization for rapid and operational analysis. In addition, in-depth studies of the GBM microenvironment performed in human tissue are limited and many conclusions are drawn from animal models or in vitro simulations [26, 34]. Therefore, high-resolution microstructural studies of patient samples are critical to understand cellular composition as well as how the tumor microenvironment is established in its real scenario. To do this, it is crucial to consider the three-dimensional architectural preservation of the human tissue including, not only the parenchyma but also the vascular niche so as to understand the full complexity of the tumor.

Thorough studies in GBM mouse models have shown that the association of glioma cells and blood vessels is essential for tumor-mass spread [10, 32], thus, special attention should be paid to analyzing the cellular properties of vascular networks in GBM patient samples. Studying the elements of the blood brain barrier (BBB) or blood tumor barrier (BTB) in human samples is of critical importance, as these may be relevant to designing new therapeutic approaches [6]. The integrity of the structural walls of the BBB could well have an impact on treatment-strategy effectiveness [1], as the degree of BTB permeability could interfere with blood cell infiltration and contribute to the cellular conformation of the tumor. In particular, the GBM stroma, as opposed to the tumor nest, appears to be composed mainly of microglia and macrophages [7], but whether this occupies a determined spatial location within the tumor mass remains obscure and needs to be determined.

Actually, both the extent to which the BTB is structurally altered [30] and how this may hamper immune cell infiltration are still debated [19]. Therefore, further studies are necessary to understand the structural and cellular relationships of BTB features in human glioma [6] and how these properties may correlate with tumor aggressiveness and patient prognosis.

We present here a unique, multicolor, high-resolution 3D view of human GBM microenvironments. We provide an atlas-like set of images and data using a highly detailed procedure that enables visualization of the cellular composition of human tumor microenvironments in relation with neovasculature and the corresponding analysis of numerical and volumetric parameters. In analyzing the BBB or BTB structural walls, we display a vascular map of GBM as well as its altered relationship with protoplasmic astrocytes and analyze the fenestration of both endothelium and basement membrane. Furthermore, we examine the permeability to lymphocytes and analyze the myeloid landscape, categorizing a distinct histocytometric glioma-associated microglia and macrophages (GAMM) profile for locating stroma and tumor nest.

## Materials and methods

### Human GBM samples

In the present study we analyzed three-dimensional biopsies (3DBx) to spatially explore the tissue and overcome the limitations of regular 5–10 μm thin sections normally used for neuropathology and diagnosis. To do so, a special protocol was set up to obtain optimally preserved volumetric sections. Human GBM samples were excised surgically at the Valencia General Hospital. Tumors were removed and prepared following the protocol previously described by us [2] and approved by ethics committees of the institutions involved. It should be emphasized that tumors were excised and immediately fixed, cryoprotected and sectioned in a cryostat (Leica Microsystems, Wetzlar, Germany) thus preventing any significant tissue autolysis and preserving intact structure, an essential requirement for the type of analysis described herein. Tumors were graded in-hospital and the Ki67 labeling index was assigned by the pathology department. All tumors studied and imaged as part of this study were grade IV glioma or GBM with Ki67 indices ranging from 10 to 50% (Additional file 1: Supplementary Table S1) to compare with normal cortex (CTX). For nontumoral CTX, we used a normal brain from an individual who died from nonpathological causes. In this case, we were able to select the frontal CTX, paralleling the area where GBM are mostly located, and cut the tissue in thick sections following the same protocol as for tumor samples.

Working with thick sections was meticulous. It required great care and significantly longer working times to preserve intact tissue structure, achieve full stain penetration and coverage and later acquire sufficient image data. The method followed and described herein necessitated a continual balance between feasible and optimal processing times to ensure quality results.

### Multilabeling immunohistochemistry

The 60-μm tumor sections were cut serially through the entire sample, and multilabel immunofluorescence staining was performed by free-floating using various combinations of primary antibodies (Additional file 1: Supplementary Table S2) after testing and selecting optimal antibodies for each marker.

Secondary fluorescent antibodies (Additional file 1: Supplementary Table S2) diluted in 1% horse serum (HS) were used accordingly with the host of the primary antibodies and 4,6-diamidino-2-phenylindole (DAPI) (1:1000, Invitrogen, Carlsbad, CA, USA) was used to stain nuclei. Immunohistochemical detection methods were previously optimized to achieve full and homogenous antibody penetration of the whole sections while substantially sparing tissue damage and keeping structural relationships intact, something absolutely critical to perform the analyses undertaken in this research.

The full immunohistochemistry (IHC) protocol spanned four days and comprised antigen retrieval in citrate buffer, blocking promiscuous/unspecific binding sites with HS, marking target structures with a combination of primary and highly crossed absorbed secondary antibodies and counterstaining nuclei with DAPI. Depending on the structures or cell types to be marked and bearing in mind the need to avoid duplicating antibody species in an individual multistaining procedure, the following antibody combinations were used: 1, COL-IV/GFAP/CD31/DAPI; 2, CD31/CD3/GFAP/DAPI; 3, COL-IV/MHCII/GFAP/DAPI; and 4, CD31/Iba1/GFAP/DAPI.

Incubation for 48 h with primary antibodies was followed by 24 h of incubation with the appropriate secondary antibodies. After washing, sections were incubated with DAPI in solution for 30 min. The sections were washed again, mounted on non-gelatinized glass slides and cover-slipped with antifading reagents (ProLong^®^ Gold antifade, Invitrogen; Carlsbad, CA, USA and Fluoprep reagent, bioMérieux SA, F-69280 Marcy l’Etoile, France) and once cured, examined by confocal microscope (Leica TCS SP5 or Zeiss LSM 700, at the Universitat Autònoma de Barcelona Microscopy Service and the Institut de Neurociències, respectively).

### Confocal imaging

Images were acquired using a laser scanning confocal microscope (Zeiss LSM 700) with four laser lines (405 nm, 488 nm, 555 nm and 639 nm), three objectives (Plan-Apochromat 20x/0.8, Plan-Apochromat 40x/1.3 oil DIC and Plan-Apochromat 63x/1.4 oil DIC) and appropriate image acquisition software (ZEN2010, Carl Zeiss AG, Germany).

Images for large photo mosaics were acquired with a confocal microscope with motorized stage (Leica TCS SP5), five laser lines (458, 476, 488, 496 and 514 nm), six objectives (Plan-Apochromat 10x/0.4 CS, Plan-Apochromat 20x/0.7 CS, Plan-Apochromat 40x/1.25-0.75 oil CS, Plan-Apochromat 63x/1.4-0.6 oil CS, Plan-Apochromat 63x/1.4-0.6 glycerol Corr CS and HC PL Fluorar 50x/0.80) and LAS AF software (Leica Microsystems CMS GmbH, Germany).

### Image processing

After acquisition, 3D stacks were processed for deconvolution. This image processing technique was performed to restore the imaged objects usually degraded by blurring and noise, artifacts inherent to any image acquisition system. The degree of blurring of a single, sub-resolution point-like object was considered as a measure of the optical system’s quality and the blurred 3D image of this single point light source was defined by the Point Spread Function (PSF) [11]. Therefore, to counteract the effects of this blurring and to optimize image quality and subsequent quantifications, all images were processed using image processing software package (Huygens Professional, Scientific Volume Imaging b.v., Hilversum, The Netherlands) or image deconvolution software (AutoQuant X3, Bitplane AG, Zurich, Switzerland).

Once images were processed to maximize quality, the relevant parameters were measured using image visualization and analysis software (Imaris 8.3.4, Bitplane AG, Zurich, Switzerland; Fiji ImageJ, Bethesda, Maryland, USA; IllucidaFX, Los Angeles, USA). Full descriptions of methodology and parameters studied are given in the subsequent sections.

### Parameter characterization and quantification

The parameters measured included total cellularity; total units (indivisible cell-size particles expressing a given marker), volumetric surface area (external surface area of an isosurface) and volume (occupied by an isosurface) in tissue blocks. Specifically measured were volumetric surface area and volume of GFAP; volumetric surface area and volume of COL-IV, blood vessel calibers; vessel branching types and totals; vessel wall fluorescence variations; volumetric surface areas and volumes of CD31-positive endothelial cells; overlapping of CD31 and COL-IV surfaces; Iba-1-positive units, volumetric surface areas and volumes; MHCII-positive units, volumetric surface areas and volumes and overlapping of MHCII and GFAP structures; and total T cells (expressing CD3) and their contact with or distance from blood vessel endothelium.

### Exploration and quantification methodologies

In line with the various research objectives of this study to closely examine the topographical and morphological relationships of human GBM microenvironment components in 3D and compare them across tumors with normal cortical tissue, we adopted the following strategy. First, we examined overall cellularity, which is known to increase in GBM. We then centered on parameters described above for GFAP expression, as this is a canonical marker for GBM diagnosis. Next, given the fundamental role of vascular networks in GBM and close relationship with GFAP cells, we performed a detailed analysis of BVs including separate COL-IV and CD31 parameters and surface overlap and blood vessel characteristics listed above. Microglia and macrophage populations were then examined for Iba-1 and MHCII expression and contiguous MHCII and GFAP surfaces. Total T cells were counted, and T cell distribution was calculated along with distance from nearest BVs. As a final component of our overall strategy, individual parameters were analyzed statistically, and Pearson correlation coefficients were calculated comparing individual parameters among themselves. Details of each step in this strategy are presented in the following sections.

### Cellularity

Dense cellularity is characteristic of gliomas and measuring this parameter in the current context served a two-fold purpose: to corroborate the Ki67% indices assigned to the tumors when excised and, depending on the concordance of our cell counts with the hospital-designated Ki67% index, to indirectly validate our analytic procedure. It also permitted us to examine how total cell count varied with the Ki67% index and other parameters. Spherical isosurfaces coinciding with individual cells identified in each sample as DAPI-stained nuclei were generated to estimate the total cell numbers (Additional file 2: Supplementary Figure S1) (Imaris, “Spots” module).

### GFAP area and density

**Fig. 1:**
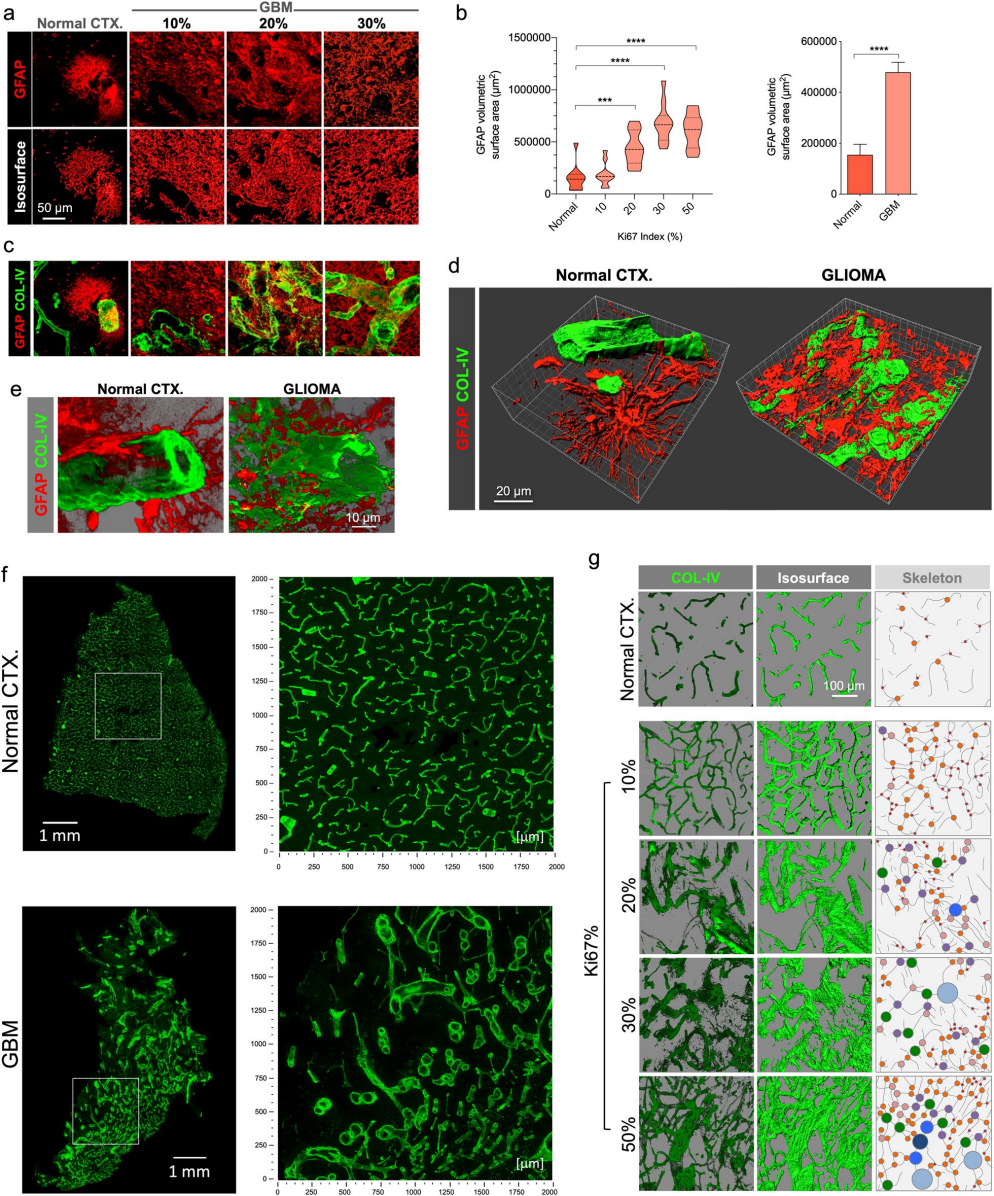
Vascular map and GFAP’s altered spatial relationship in GBM. **a** GFAP distribution of characteristic protoplasmic astrocytes in normal CTX, and glioma cells in GBM-affected cases, displayed on original confocal images (top panel) and the corresponding rendered isosurfaces (bottom panel). **b** GFAP-expressing cells show a massive increase in GBM as reflected in volumetric surface area analysis relative to Ki67% and all tumor cases grouped. **c** Visualization of correlative vasculature associated with GFAP^+^ cells in normal brain and GBM. Merged views of GFAP from panel A, and blood vessels, evidenced by COL-IV (green) are shown. **d** 3D rendering of GFAP and COL-IV in normal CTX and GBM highlight the differences between normal and GBM blood vessels in relation with surrounding GFAP-expressing cells. **e** Detailed, higher magnification 3D renderings show altered spatial relationship of basement membrane and GFAP. **f** Hypermosaic of cortical sample of a normal brain CTX and GBM, and high-resolution mosaics from white inset. **g** Confocal alpha blending, 3D rendering of high-magnification images and skeletonization (including branching points color-coded by size) reveal dramatic changes in blood vessel distribution and architecture relative to increasing Ki67 index in GBM

GFAP volumetric surface area and contained volume were measured in confocal images by importing them into 3D visualization software and creating 3D isosurfaces (Fig. 1) (Imaris, “Surfaces” module).

### Collagen IV area and density

We quantified the amount of COL-IV in confocal images by importing them into 3D visualization software and creating 3D isosurfaces based on detected fluorescence (Fig. 1) (Imaris, “Surfaces” module). Statistics for total surface area, total volume were generated by the software and exported as CSV files for calculations and statistical analysis.

### Vessel diameter

**Fig. 2:**
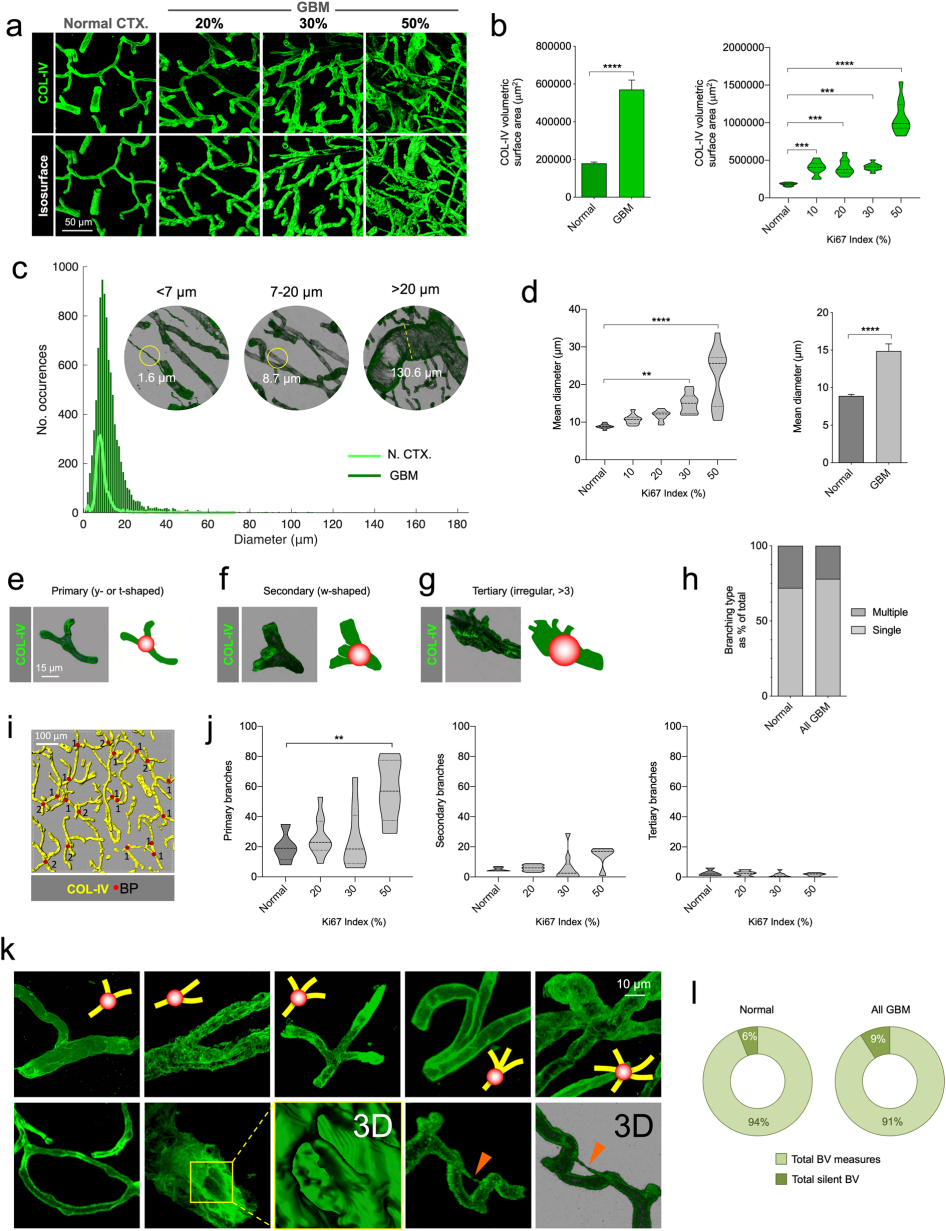
Volumetric increase of GBM vasculature and heterogeneity is due to increased calibers and primary branching. **a** Creation of COL-IV isosurfaces in normal CTX and GBM. **b** Volumetric surface increases in GBM. **c** Measurements of diameter of COL-IV^+^ vessels in normal CTX (N. CTX) and GBM. **d** Quantifications of diameters in GBM compared to normal CTX. **e**, **f** and **g** Alpha blending of primary, secondary and tertiary branching points. **h** Percentage of branching types (single and multiple) in normal CTX and GBM. **i** Representative image of isosurface rendering and quantification of branching points. **j** Quantification of primary, secondary and tertiary branching points. **k** Representative images of branching points in GBM from primary to tertiary (top panel), and looping, intussusception and silent collateral vessels (bottom panel, from left to right respectively). **l** Quantification of silent collateral BV in normal and GBM

To assess vessel diameters, 3D confocal images of tissue blocks were imported into image analysis software and the widths of all visible vessels in each image were measured (Imaris, “Measurement Points” tool) (Fig. 2). Measurement data were exported to additional software to calculate statistical parameters (CSV file in MATLAB [Natick, Massachusetts, USA]).

### Branching

To quantify and categorize branching, vessel ramifications were observed three dimensionally and divided into primary (y- or t-shaped single bifurcations), secondary (w-shaped with two ramifications) and tertiary (irregular multi-branching with three or more ramifications) (Fig. 2). Confocal images were imported into the appropriate software (Fiji distribution of ImageJ) and the total single and multiple branches in each image were counted using unbiased stereological criteria [21].

### Vessel wall integrity

**Fig. 3:**
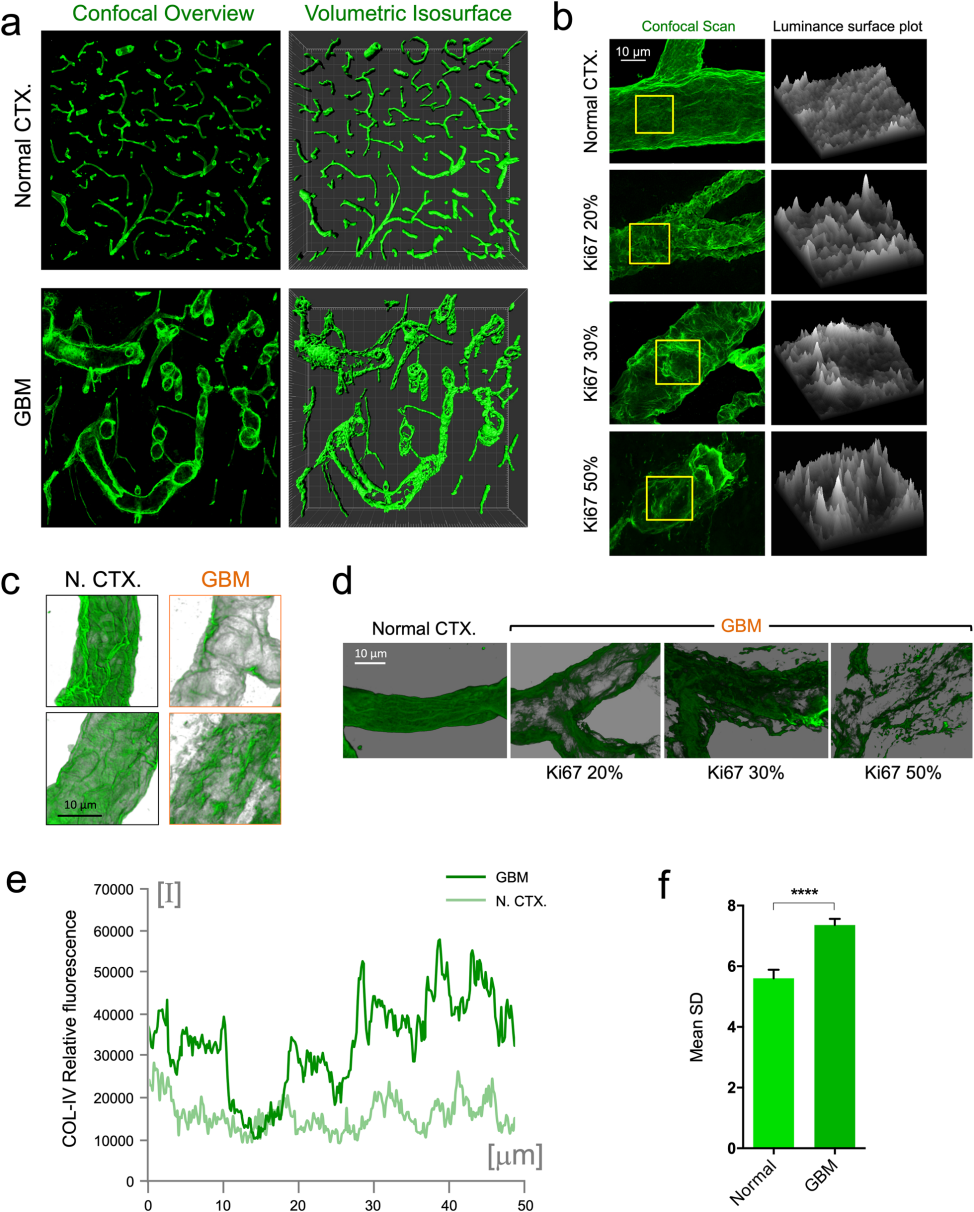
GBM BVs show uneven COL-IV distribution reflecting basement membrane disruption and fenestration. **a** Comparison of high-resolution confocal mosaic in normal CTX and GBM. Original confocal overview and volumetric isosurfaces are shown (large increments in frame are 50 μm). **b** Luminance surface plot measured along the basement membrane of normal CTX vessel and GBM-associated vessels according to the proliferation index Ki67. **c** Contrasted transparency of basement membrane in normal CTX (N. CTX) and GBM. **d** Aspect of altered COL-IV distribution in GBM according to increasing Ki67. **e** Measurements of relative basement membrane fluorescence in normal CTX (N. CTX) and GBM. **f** Standard deviation of relative fluorescence of normal CTX and GBM basement membrane

To quantify the vessel surface disruption, irregularities and fenestrations, we measured variations in fluorescence intensity along small segments of vessel walls assuming that vessel wall continuity and integrity would be reflected indirectly in this variation. Single 1-μm optical slices, were exported from confocal image stacks and small segments of five vessel walls in each sampling image were measured by tracing 40-μm lines along blood vessel walls using ImageJ software to generate a series of values for fluorescence levels along the respective lines (ImageJ, “Plot Profile” function). Individual line lengths varied slightly but the profiles were harmonized after exporting by only using the fluorescence intensity values corresponding to the first 27 microns measured for each sample (Fig. 3).

### CD31 area and density

To quantify any changes in CD31 expression relative to increasing tumorigenicity, analogous to the basement membrane analysis, we captured confocal images and imported them into image analysis software (Imaris, “Surfaces” module) to create 3D isosurfaces and then exported the isosurface areas and volumes for calculations and statistical analyses.

### Surface overlap between CD31 and collagen IV

In intact blood vessels, the vascular basement membrane is lined with endothelial cells. To examine this, images were acquired from samples immunostained for both COL-IV and CD31 and imported into image analysis software (Imaris or IllucidaFX). Three-dimensional isosurfaces were created from the COL-IV and CD31 channels and a mask was created separately for each channel from the respective 3D isosurface to eliminate artifacts from the original confocal channels. A new channel was then generated from the overlapping structures of the masked COL-IV and CD31 channels in the confocal image stack and a 3D isosurface of this overlapping area was created. From the latter, total overlapping areas were extracted.

### Glioma associated microglia and macrophages

#### Iba-1

Iba-1 volumetric surface area, units and volume were measured in confocal image stacks by importing them into image analysis software and creating 3D isosurfaces (Imaris, “Surfaces” module).

#### MHCII

Human GBM biopsy samples were immunostained marking COL-IV, GFAP, MHCII and nuclei. MHCII volumetric surface area, units and volume were then measured in confocal image stacks by importing them into image analysis software (Bitplane’s Imaris or IllucidaFX) and creating 3D isosurfaces (“Surface” module). A separate colocalization channel was created and isosurfaced to measure the total surface area of overlapping MHCII/GFAP areas. In addition, MHCII-rich and GFAP-rich areas were isolated by selecting regions of interest (ROI) and the area and volume of COL-IV expressed in each ROI was measured. Vessel diameters were measured separately for each ROI.

#### T cells: CD3

CD3′s antigen specificity and its universal expression at all T cell developmental stages make it an ideal immunological marker to detect T cells in tissue sections. To explore T-cell populations in GBM, tumor tissue samples were immunostained for CD3 and acquired confocal images were imported into image analysis software (Imaris). The objective was to measure the extent of extravasation (i.e., movement of T cells away from vessels) and see if that bore any relation to membrane degradation in severe tumors. Total T cells in each image were counted using image analysis software (Imaris, “Spots” module) and the distances from or contacts with the nearest blood vessels were measured in three-dimensional visualization (Imaris, “Measurement Points” tool).

### Data analysis

#### Statistical analyses

Results for cellularity, vessel characteristics (diameters, branching and density) and GFAP and COL-IV volumetric surface areas and volumes derive from samples immunostained for COL-IV and GFAP and counterstained with DAPI with 50 data points (10 tissue blocks for normal CTX and 10 tissue blocks each for Ki67 indices 10, 20, 30 and 50%). Basement membrane continuity results are drawn from the same sample pool of tissue blocks with 50 data sets with each data set comprising five separate sets of over 25 luminance measurement points.

Results for CD31 surface area and multiple comparisons between cellularity, vessel continuity, COL-IV and CD31 surface areas, colocalization and GFAP surface area, derive from a sample set stained for COL-IV, CD31 and GFAP and counterstained with DAPI with 24 total data points for each variable, six for normal tissue and 18 for tumor tissue blocks (six each for Ki67 indices of 10, 20 and 30%). Again, basement membrane continuity results are drawn from the same sample pool with 24 data sets (six control tissue blocks and six each for Ki67 indices of 10, 20 and 30%) with each data set comprising five separate sets of 34 luminance measurement points.

Total T cell counts, and T cell extravasation and migration data were drawn from a sample pool immunostained for CD31, CD3 and GFAP and counterstained with DAPI. Thirty data sets were generated and analyzed (six separate tissue block areas for Ki67 10% and 12 areas each for 20 and 30%).

Data for Iba-1 and MHCII derive from two sample groups (each with six normal and 24 tissue blocks; tumors had Ki67% indices of 10, 20 and 30%). Iba-1 samples were immunostained for CD31, Iba-1 and GFAP and counterstained with DAPI. MHCII samples were immunostained for COL-IV, MHCII and GFAP and counterstained with DAPI.

For all statistical comparisons, we used an unpaired, two-tailed student t-test with Welch’s correction, or one-way ANOVA or Kruskal–Wallis test (Dunnett’s, Tukey’s, Dunn’s or Sidak’s multiple comparisons test) with *p < 0.05, **p < 0.01, ***p < 0.001, ****p < 0.0001, unless otherwise stated in the text. Data are represented as mean ± SEM. F-tests were used to examine the similarity of the variance of the compared groups, and the appropriate tests were performed accordingly.

## Results

To analyze the cellular complexity of brain tumor microenvironments, GBM samples obtained from surgical resection were rapidly processed to preserve the tissue structural integrity. Then, after cryoprotection, samples were cut in 60-μm thick sections (for 3D visualization) and processed for high penetration multi-labeling immunohistofluorescence. High-resolution 3D scans were captured in x–y-z planes covering the section thickness. Systematic 3D sampling of biopsies was also done to build hypermosaics. Three-dimensional stacks were deconvoluted and rendered in appropriate software to generate 3D visualizations to analyze numerical and volumetric parameters as well as cellular topographical relationships (Additional file 3: Supplementary Figure S2).

We performed an extensive 3D mapping of GBM samples at cellular resolution using multilabeling staining for vascular structures and tumor-cell types covering both peritumoral areas and tumor cores. Only a selection of images is displayed in this resource paper, and original image files in .lsm, .lif or.ims format can be examined on request.

Prior clinical neuropathological examination of the samples had determined the classical GBM features including pluricellularity, formation of hypoxic pseudopalisades, aberrant mitoses, and glomeruloid vessels. Importantly, expression of classical GBM markers GFAP and Vimentin was clearly detected in the 3DBx. Moreover, canonical gemistocytic formations were also identified on examination at high-resolution (Additional file 2: Supplementary Figure S1). Before proceeding with further in-depth imaging and to validate the homology and consistency of the clinical neuropathological assessment, full sets of confocal images were evaluated histopathologically with our automated tools to corroborate a correlative increase in cellularity with respect to increasing Ki67 index (Additional file 2: Supplementary Figure S1).

### GFAP map and expression

Using GFAP expression, one of the most accepted GBM markers in histopathology, GBM-affected cortical areas were mapped and tumor location was made evident given the distinct morphology of resident astrocytes in different cortical layers and the tumor mass itself surrounded by layers of reactive astrocytes (Additional file 4: Supplementary Figure S3). Tumor mass could be clearly detected as a heterogeneous and disorganized bulk of cells with shapes ranging from fibrous to swollen-eccentric gemistocytic formations. Sampling of GBM 3DBx in cases with increasing Ki67 indices showed higher GFAP density and an increase in the volumetric surface area of the GFAP^+^ elements (Fig. 1a and b). Close proximity to interlaminar and varicose-projecting astrocytes served as a hint to histologically locate the tumor, frequently hosted in deeper layers of the CTX and consistent with the potential origin described in previous studies [14].

### Basement membrane vascular network

To visualize the blood vessels, staining collagen IV (COL-IV)-rich basement membrane provided a clear and reliable outline of the vasculature both in control CTX and GBM (Fig. 1c–g). In some GBM cases, there was massive COL-IV deposition, in some rare areas with barely recognizable vessels (Additional file 5: Supplementary Figure S4).

Visualizing the vascular 3D footprint revealed not only the complexity of interacting structures in normal CTX but also the altered spatial relationship between basement membrane and GFAP^+^ cells in GBM (Fig. 1d). GFAP^+^ cells in GBM appeared as a dense mass with variable morphology with irregular fibrillary processes and gemistocytes (Additional file 4: Supplementary Figure S3 and Fig. 1d and e) in contrast with regular protoplasmic astrocytes in normal CTX. GFAP^+^ glioma cells were often in apposition and surrounding BVs, compatible with the phenomenon of tumor cell motility along BV via the outer side of the basement membrane, events that are compatible with previous observations seen in glioma mouse models [32, 33]. This blood vessel-GFAP cell interaction was also seen in pseudopalisades, being fibrous in the hypercellular areas (Additional file 6: Supplementary Figure S5), consistent with the escaping behavior of GBM cells in these structures [3, 29].

To map and understand the vascular network, we built three-dimensional hypermosaics that exhibit the heterogeneity of GBM vasculature compared to unaltered normal CTX. Non-neoplastic CTX appeared consistently irrigated with a regular vessel network. In contrast, GBM showed uneven vessel distribution with irregular vessel shapes (Fig. 3f). 3D rendering and shadowing allowed correct visualization of this heterogeneity, which increased in accordance with the Ki67 index (Fig. 1f and g), which demonstrates in tissue that the impaired structural adaptation and transformation of tumor-induced angiogenesis, predicted by computational simulations [25], correlate with malignancy.

By generating 3D isosurfaces, we were able to measure COL-IV volumetric surface area, demonstrating an increase in volume in GBM, especially in highly proliferative cases (50% Ki67) (Fig. 2a and b). As this increased heterogeneity may be due to an increase in blood vessel caliber, we analyzed the occurrences of blood vessels in three diameter ranges, smll, medium and large, and found vessel diameters were increasingly larger in more proliferative tumors (Fig. 2c and d).

Because this heterogeneity could be due to the generation of new ramifications, we also analyzed and quantified vessel branching. To do so, we arbitrarily categorized branching type in 3D images according to the number of branches emanating from each branching point sampled [primary (y- or t-shaped), with two branches; secondary (w-shaped), with three branches and tertiary (irregular, > 3), with four or more branches]. Interestingly, branches generated were mostly of the primary type whereas secondary and tertiary were both minimal (Fig. 2e–j). These results suggest that the major strategy for generating new vessels in human GBM is single branching, in contrast with other potential strategies [4, 8].

Because angiogenesis, besides sprouting, can be accomplished by several alternative strategies, such as looping, splitting (intussusception) or activation of silent collateral vessels [12], we acquired images of these structures from GBM 3DBx for analysis. We imaged arrangements that were consistent with looping and also with the formation of COL-IV pillars compatible with intussusception (Fig. 2k). We also observed numerous silent collateral vessels (Small vessels ≤ 5 μm diameter insufficient to accommodate blood cell passage) although quantification of the latter did not resolve whether or not this strategy is used, as the proportion appears to be similar in GBM and normal CTX (Fig. 2l).

High resolution, in-depth imaging and 3D rendering, and shadowing of COL-IV revealed a patent structural difference between normal and GBM-affected CTX (Fig. 3a). Healthy CTX displayed a regular vascular network with uniform appearance of COL-IV, whereas GBM-affected CTX showed a heterogeneous distribution and disrupted basement membrane (Fig. 3a) with a fenestrated appearance. Analysis of fluorescent-labeled COL-IV luminance showed uniform levels in normal CTX while GBM vessels displayed drastic peaks and valleys (Fig. 3b). Alpha blending of COL-IV images shows the disparity between the crêpe-paper-like appearance of normal vessels and the disrupted aspect of GBM vessels (Fig. 3c). Interestingly, this COL-IV disruption appears to be more dramatic in more proliferative tumors (Fig. 3d). Analysis of relative fluorescence provided clear evidence of this feature (Fig. 3e), showing a significant increase in relative fluorescence variation possibly indicating microscopic disruption of the basement membrane in human GBM (Fig. 3f).

### Endothelial vascular network

**Fig. 4:**
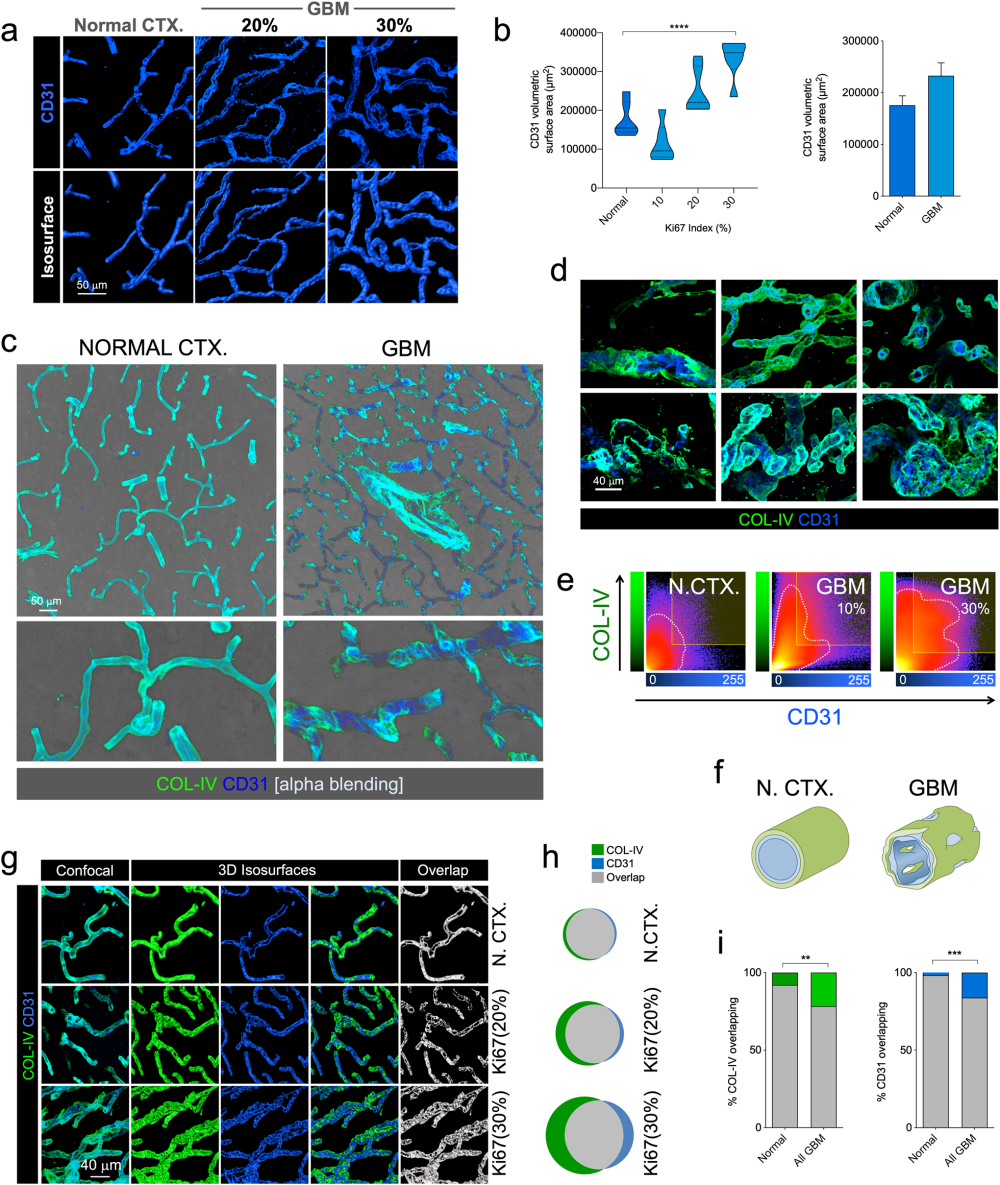
GBM endothelium appears disrupted from the basement membrane resulting in BTB multilayer fenestration. **a** Confocal images and isosurface renderings of CD31^+^ endothelium for normal CTX and two GBM cases with 20 and 30% Ki67 indices. **b** Increased volumetric surface in high Ki67% cases. **c** Alpha blending of COL-IV and CD31 in normal CTX and GBM. Overview is shown in top panel and higher magnification in bottom panel. **d** Detail of different vascular structures in GBM labeled with COL-IV and CD31. E. COL-IV and CD31 colocalization plots in normal CTX (N. CTX) and GBM with 10 and 30% Ki67. F. Diagram of the basement membrane and endothelium in normal CTX (CTRL) and GBM. **g** Confocal images of COL-IV and CD31, 3D isosurface rendering and surface overlap in normal CTX and two cases of GBM with 20 and 30% Ki67. **h** Venn diagrams showing the 3D surface overlap of COL-IV and CD31 in N. CTX and GBM cases. **i** Percentage of overlapping COL-IV and CD31 3D surfaces in normal CTX and GBM-affected CTX

Because basement membrane was seen as highly fenestrated, we analyzed whether this phenomenon occurs equally in the adjoining endothelium. To do so, we labeled the endothelium with CD31 (Fig. 4a), a reliable marker, paralleling previous vascular measurements made in glioma mouse model [32], and quantified the volumetric area of the vascular network (Fig. 4b). We saw that endothelium increases in highly proliferative tumors, but the global increase was of a lesser extent than that of the basement membrane. Double labeling of basement membrane and endothelium in GBM-affected cortices showed that the two did not seem to match completely, in contrast with the regular overlap seen in normal CTX (Fig. 4c and Additional file 7: Supplementary video 1). Particularly, two different scenarios could be observed in GBM cases, COL-IV visualized without overlapping endothelium, and endothelium seen without overlaying basement membrane (Fig. 4c, d and Additional file 8: Supplementary Video 2 and Additional file 9: Supplementary Video 3). In some cases, endothelial cells were highly proliferative, resembling the first stages of glomerulus formation, and in other cases occupying canonical glomeruloid vascular tufts in large numbers with no corresponding neoplastic cells (Additional file 10: Supplementary Figure S6 and Additional file 11: Supplementary Video 4). Further analysis of the overlapping voxels between COL-VI and CD31 isosurfaces revealed a homogeneous interaction in normal CTX, with almost 100% isosurface overlap compared with tumor-bearing CTX (Fig. 4e–g). Detailed quantification of the interacting 3D isosurfaces demonstrated an intimate interaction in control CTX in contrast with an increasingly disrupted one in GBM cases (Fig. 4h and i).

As we had observed dramatic fenestration of endothelium and basement membrane in GBM compared to normal CTX, we were interested in examining its relationship with the external wall of the BBB or BTB by labeling astrocytes or glioma cells with GFAP. This becomes especially relevant considering that previous studies in glioma mice models showed that astrocytic endfeet are displaced by glioma cells during the co-option process through initial tumor growth [32].

**Fig. 5:**
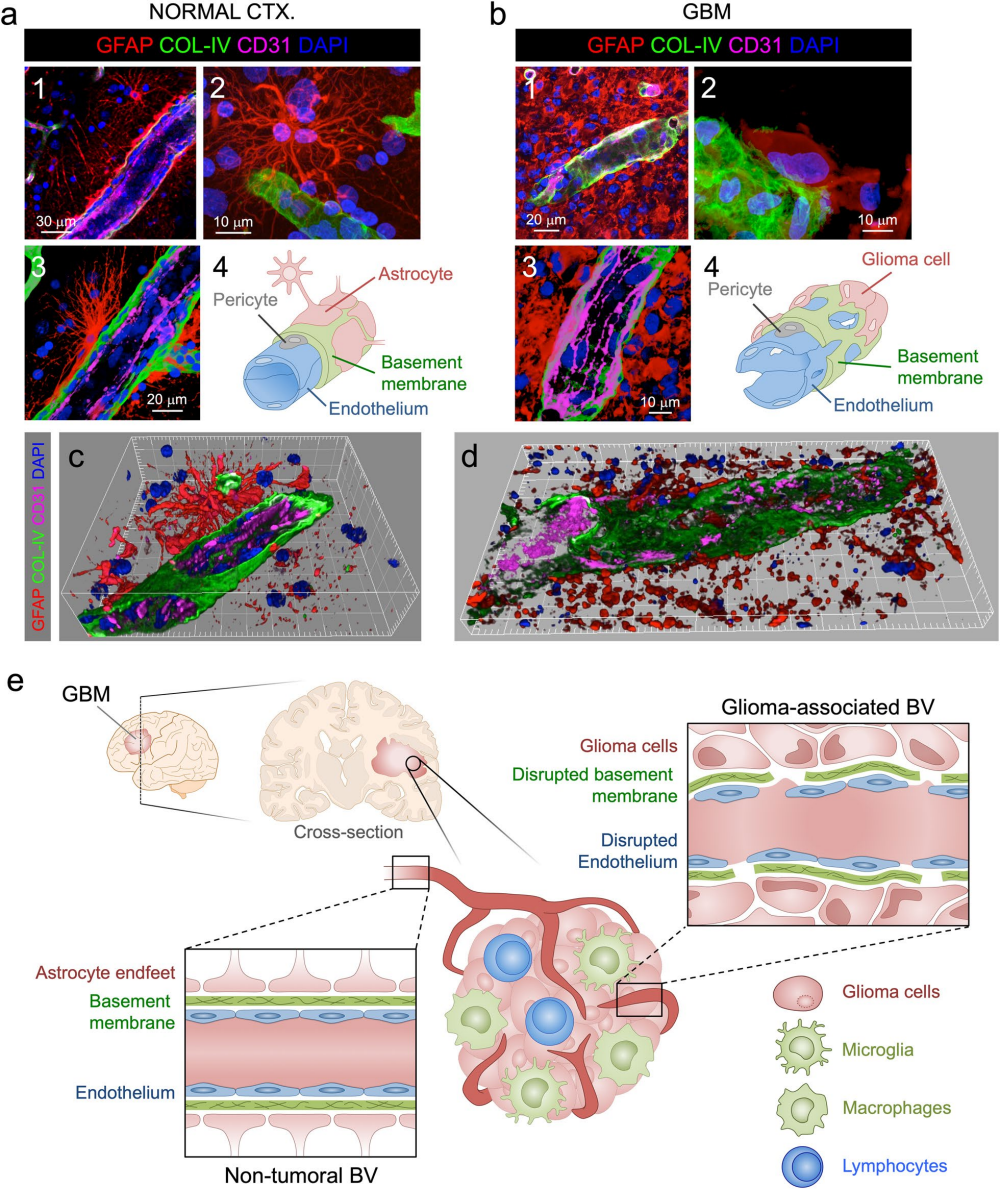
Disrupted BTB structural elements in GBM. **a** Characteristic structure of brain BV elements in normal CTX. Overview of the architectural integrity of brain BV formed by protoplasmic astrocytic endfeet, COL-IV basement membrane and CD31 endothelium (1). 3D detail of a BV-related protoplasmic astrocyte (2). Higher magnification of the brain vessel integrity (3). Diagram of the normal CTX BBB elements (4). **b** Characteristic disrupted structure of brain BV elements in GBM. Overview of the altered vascular structure in GBM gemistocytic tumor nest (1). Detail of classical GBM gemistocyte on disorganized basement membrane (2). Detail of the intermingled elements of the GBM associated vessels (3). Diagram of the GBM BTB elements (4). **c** 3D reconstruction of a normal cortical BV (Large increments in frame are 5 μm). **d** 3D reconstruction of a GBM-associated BV (Small increments in frame are 5 μm). **e** Diagram illustrating the alterations in GBM-associated BV versus non-tumor BV

Combined labeling of three primary BBB components, endothelium, basement membrane and astrocytes or glioma cells (Fig. 5a and b) and detailed high-resolution 3D reconstruction of normal CTX and GBM (Fig. 5c and d), depicted complex, multilayered fenestration in GBM compared to the regular and continuous structure in normal CTX with clear variations in GFAP layers (Fig. 5c–e). In normal CTX, protoplasmic astrocytes were seen in close apposition to homogeneously built vessels (Fig. 5a, c and e) whereas GBM cases showed gemistocytic glioma cells coopting the external walls of disrupted vessels (Fig. 5b, d and e) where anomalous, not easily discernible GFAP-expressing astrocytic-like structures were recognized.

#### T cell infiltration and migration

**Fig. 6:**
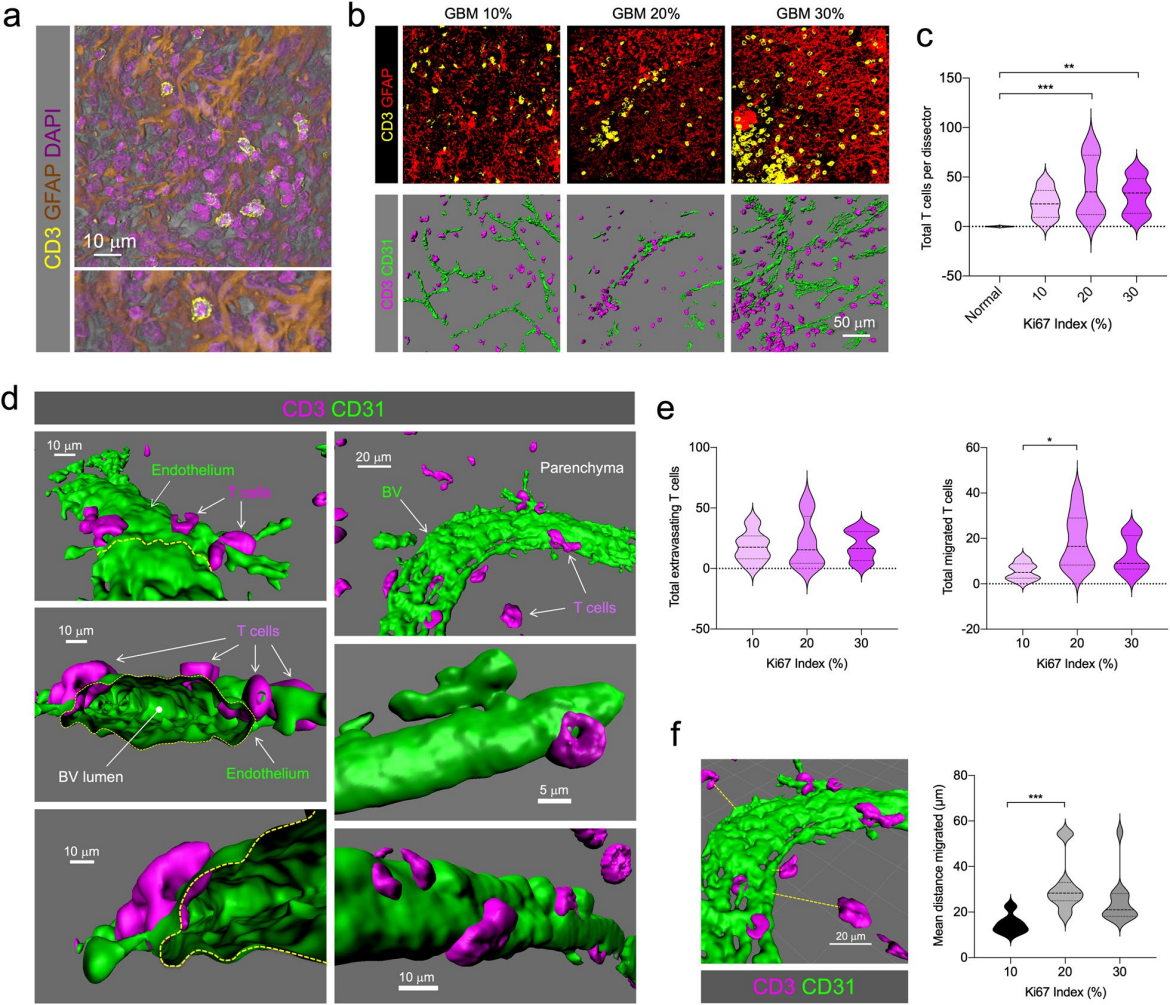
Increased T cell infiltration and migration in GBM. **a** Representative T cell infiltration in GFAP^+^ areas. Higher magnification of a CD3^+^ T cell is seen in the bottom panel. **b** Representative images of CD3^+^ T cell infiltration (yellow) and GFAP expression (red) in GBM patients with 10, 20 or 30% Ki67 indices as determined by neuropathological examination. Bottom panel shows the rendering of T cells (magenta) and CD31^+^ endothelium (green). **c** T cell quantification in GBM patients. **d** Three-dimensional analysis of T cell position with respect to endothelium distinguishes extravasating T cells (in diapedesis) or migrated into the tumorigenic parenchyma. **e** Quantification of extravasating and migrated T cells. **f** Quantification of the distance travelled from the closest endothelium

As the multilayered fenestration described above may affect immune cell permeation of tumor parenchyma, a recent case in point [19], we analyzed T cell homing or infiltration in GBM 3DBx. T cell presence was detected by labeling CD3, and combined with endothelium marker CD31, GFAP to detect the tumor mass, and DAPI counterstaining to identify individual cells (Fig. 6a and b). T cells and vascular endothelium were segmented and specific 3D isosurfaces were generated to perform quantifications. Total number of T cells was increased in all GBM cases, compared to normal CTX, but no differences were seen between groups according to Ki67 index (Fig. 6c). High-resolution analysis allowed the precise detection of T cells attached to the endothelium (extravasating) or infiltrated into the tumor mass (migrated) (Fig. 6d). This allowed us to analyze three dimensionally the distance T cells travelled from the endothelium into the tumor. The number of extravasating T cells was similar in all tumor cases suggesting an inflammatory-mediated lymphocyte attraction and initiation of diapedesis. However, the number of migrated cells appears to increase in more proliferative tumors (Fig. 6e). Interestingly, the distance traveled appears to increase in tumors with higher proliferation index (Fig. 6f).

### Mapping the myeloid landscape

**Fig. 7:**
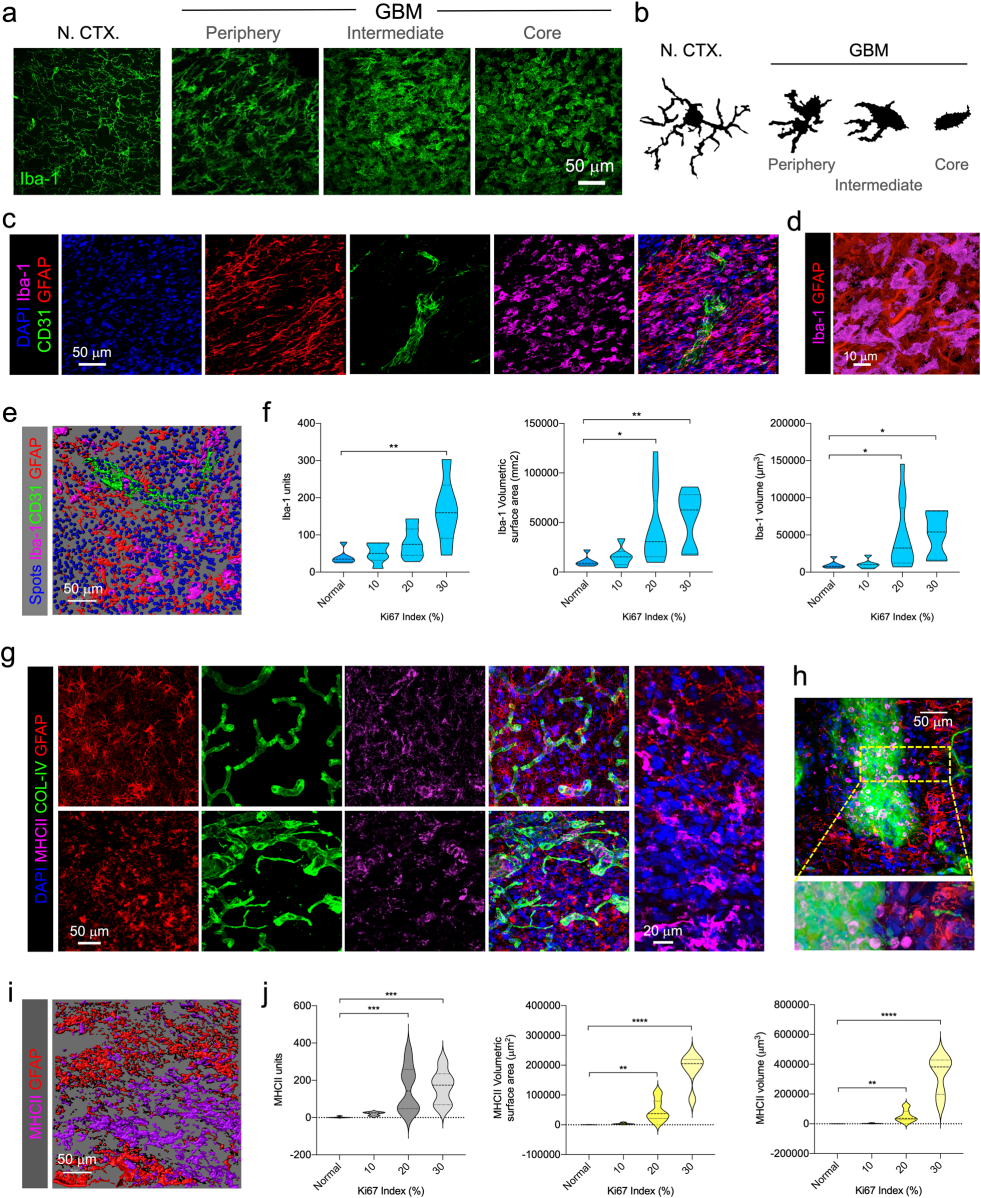
Myeloid landscape in GBM. **a** Morphological spectrum of GAMMs in GBM compared with cortical microglia in normal CTX. Tumor periphery, intermediate areas and core are shown. **b** Computerized camera lucida of characteristic morphological spectrum of GAMMs. **c** Iba-1^+^ GAMMs (magenta) in GBM tumor core (GFAP) in proximity to BV (CD31). **d** Higher magnification of Iba-1^+^ GAMMs (magenta) in GFAP-rich (red) area. **e** Rendering of Iba-1 (magenta), CD31 vessels (green) and GFAP (red) isosurfaces, together with cell-size DAPI spots (blue). **f** Quantification of Iba-1^+^ units, volumetric surface area, and volume in normal CTX and GBM cases. **g** Visualization of DAPI^+^ nuclei (blue), GFAP^+^ cells (red), COL-IV (green) vessels, and MHCII^+^ cells (magenta) in peripheral areas and tumor core. **h** MHCII^+^ cells in close proximity to blood vessels. **i** Representative image of mutually exclusive GFAP (red) and MHCII (magenta) isosurfaces in GBM. **j** Quantification of MHCII units, volumetric surface and volume in normal brain and GBM cases

Along with T cells, monocytes also permeate the tumor parenchyma, becoming macrophages associated with the tumor and joining the resident macrophage-like population, microglia [7]. The two cell-types, are hardly distinguishable when in the tumor core and are normally pooled as GAMMs [24]. Thus, to identify GAMMs, we first used the pan marker Iba-1. Cortical microglia in normal CTX showed the classical morphology, displaying a homogeneous network of thin processes (Fig. 7a and b). In GBM samples, a gradual morphological spectrum was identified. The peripheral rim of the tumor showed layers of activated GAMMs, with morphologies compatible with a pre-activated phenotype with larger cell bodies and thick branches. By contrast, in the tumor core GAMMs showed fully activated shapes without branches and with a rounded cell-body outline (Fig. 7a and b). Intermediate zone GAMMs displayed transitional shapes. Throughout the tumor, Iba-1^+^ GAMMs appear distributed in all niches, with no distinctive or particular association, i.e. located either near or distant to blood vessels (Fig. 7c–d). Quantification of Iba-1^+^ GAMMs, using 3D isosurfaces revealed an increase corresponding with the Ki67 proliferation index (Fig. 7e and f), which is concordant with the idea that GAMMs may contribute to tumor survival.

To further characterize pro-inflammatory GAMMs, we used MHCII (HLA-DR) immunofluorescence, a marker classically used for activated states of microglia and brain macrophages [17]. MHCII was detected in peripheral areas and tumor core, showing particularly strong intensity in perivascular areas (Fig. 7g and h) in close apposition to the basement membrane, compatible with perivascular macrophages. Certain areas displayed MHCII^+^ cells homing through permeable blood vessels (Fig. 7h). A similar phenomenon could also be seen in vessels surrounded by necrotic pseudopalisades (Additional file 6: Supplementary Figure S5e). Quantification of MHCII expression revealed increasing numbers in more proliferative tumors and interestingly the areas occupied by MHCII were particularly elevated in tumors with the highest Ki67 Index (Fig. 7j).

**Fig. 8:**
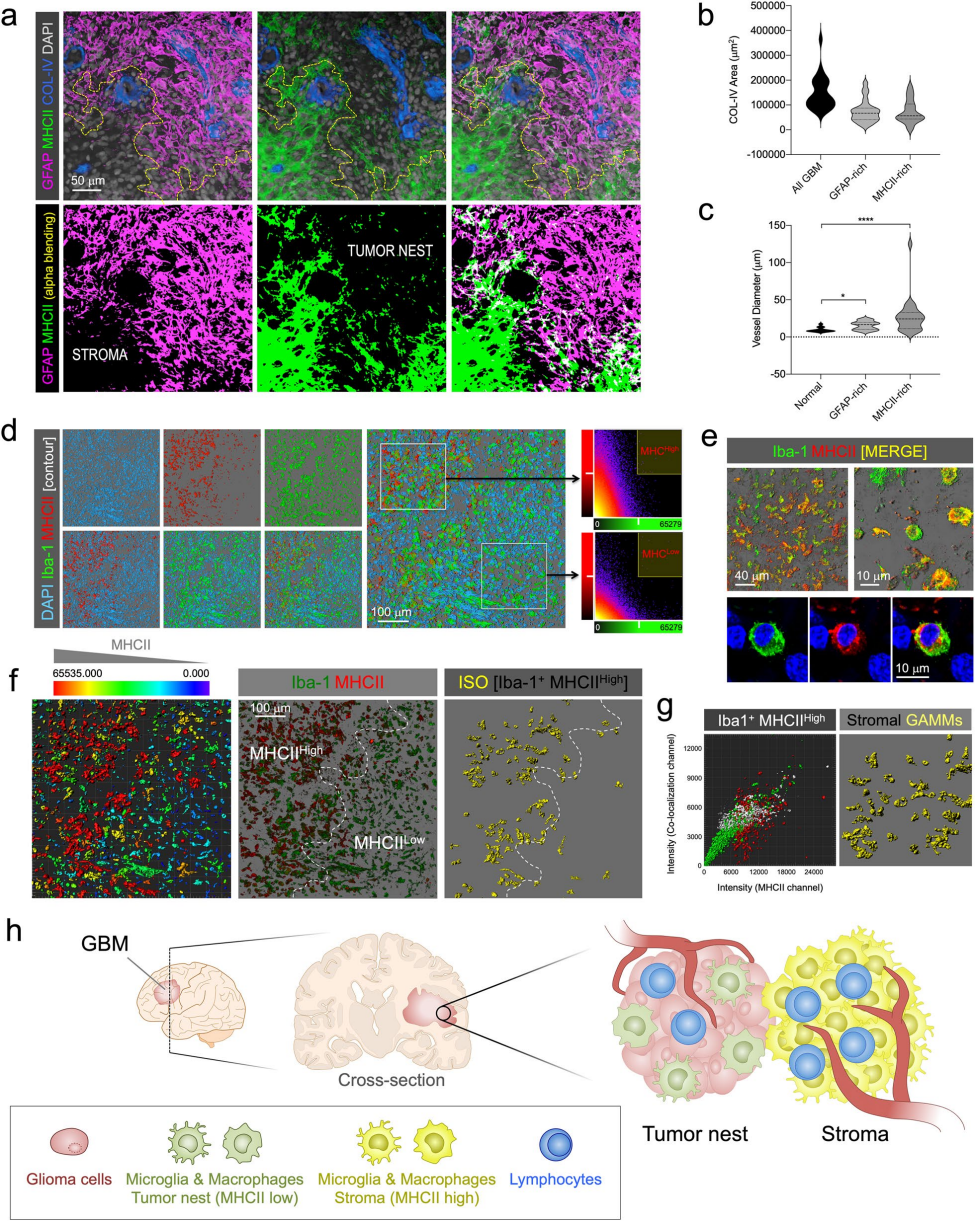
Distinct neuro-inflammatory profile of GAMMs in stroma and tumor nest. **a** GFAP (magenta) and MHCII (green) label tumor nest and stroma, respectively, as mutually exclusive niches in GBM. COL-IV blood vessels (blue) and cell nuclei (gray) are also marked. **b** Vascular volume of stroma (MHCII-rich) and tumor nest (GFAP-rich) suggested equal perfusion. **c** Vessels in stroma (MHCII-rich) have larger calibers than in tumor nest (GFAP-rich). **d** Segmentation by contour of MHCII (red) and Iba-1 (green) cells from a GBM confocal stack. DAPI was used as counterstain (cyan). Fluorescence intensity of the green and red channels plotted in MHCII^High^ and MHCII^Low^ profiles. **e** Top panels show confocal alpha-blended images of Iba-1 (green) and MHCII (red) labeling combination in GBM samples to visualize colocalizing cells (yellow: colocalization). Bottom panels show a high-resolution optical slide of an Iba-1/MHCII-expressing GAMM. DAPI (blue) was used for counterstaining. **f** Categorization of segmented MHCII^+^ cells according to MHCII intensity. Selection of segmented Iba-1^+^ with high intensity of MHCII. **g** Left. Cytohistometry graph of Iba-1^+^ (green) and MHCII^+^ (red) cells showing the selection of Iba-1^+^/MHCII^High^ (white) as stromal GAMMs. Right. Selected cells highlighted in the image (yellow). **h** Diagram of the GAMM neuroinflammatory profile distribution in tumor nest and stroma

Most importantly, the expression of MHCII was low or undetectable in GFAP-expressing areas, and conversely, GFAP^+^ cells were not detected in MHCII-rich areas, showing mutually exclusive regions with no clear overlap and differentiating the tumor nest from the stroma (Fig. 8a and b and Additional file 12: Supplementary Video 5).

Both areas were equally perfused by blood vessels as COL-IV isosurfaces revealed similar volumetric parameters in each, although, the caliber of vessels within MHCII-rich regions was higher (Fig. 8c). To validate the differential expression of MHCII in GAMMs, we combined Iba-1 and MHCII labeling. Cells were contoured in confocal stacks and the level of MHCII fluorescence was sufficient to discriminate two subpopulations of Iba-1^+^ cells according to the fluorescence intensity of MHCII channel (Fig. 8d). At higher resolution, both GAMM subpopulations could be easily identified (Fig. 8e). Then, by creating cell-size isosurfaces, histocytometry plots could be generated, allowing us to sort Iba-1^+^ MHCII^High^ stromal GAMMs (Fig. 8f and g).

Stroma in GBM has scarcely been investigated, and although it is thought to be mostly composed of GAMMs [7], its topographical differentiation from the tumor nest is still unclear. This method allows the use of a marker that may separate the populations of the two environments (Fig. 8h).

Overall, this resource article provides an atlas-like image collection accompanied by precise measurements of numerical and volumetric parameters. We hope this may help to further understand human GBM microenvironment complexity and to shed light on GBM’s architectural and cellular composition, both of which should be considered for further studies or when taking therapeutic action.

## Discussion

We present here a reliable, multicolor labeling method to visualize the cellular microenvironments of GBM three dimensionally. We ruled out other standard multiplex procedures especially those requiring aggressive microwaving of samples in some steps between labeling and staining, which would have damaged the tissue structure in floating, thick 3DBx. Our images and novel analysis shed light on the architectural heterogeneity and cellular complexity of human GBM microenvironments and are made available as a resource for further studies.

Classical GFAP labeling allowed us to identify and map an array of morphological changes in GBM and relationships with blood vessels. Importantly, these changes correlate with vascular heterogeneity, which is characterized by the increase in branching points and variation of calibers.

The detailed analysis of endothelium, basement membrane and astrocytic endfeet show that the structural layers of the BBB are altered in a particular manner, displaying a multilayered fenestration. These results suggest that vascular integrity is compromised in GBM, although this may still be a matter of debate [19, 30]. Most interestingly, this basement membrane and endothelium fenestration in GBM, each with a different disruption pattern, may have important consequences in immune cell permeation.

Our analyses demonstrate that T cells home into the tumor parenchyma and remarkably, tumor proliferation seems to positively correlate with the level of migration of lymphocytes within the tumor. This actually challenges the conception of malfunctioning vessel wall and poor entry of immune cells in tumors [19], as T-cell adhesion and transmigration appear to be functional in GBM. This is actually consistent with previous results from our group, in which we showed that T cells enter the GBM parenchyma as nonproductive lymphocytes in a patrolling mode, displaying a kinaptic state, which entails increased motility and random walking within the tumor [5]. At least for GBM, the BTB does not seem to hamper immune cell infiltration. CD31^+^ endothelium in GBM appears to function properly as an adhesion layer [23] to facilitate leukocyte diapedesis (including lymphocytes and monocytes) and entry. But most importantly, the endothelial fenestrations that expose the basement membrane to circulating immune cells may also be functional. Lymphocytes can also use these open areas for extravasation since VLA-1 integrins in lymphocytes can directly bind COL-IV to facilitate their infiltration [27, 28].

Our results also show a high density of GAMMs deriving from resident and blood infiltrate monocytes. Quantifications consistently indicate that the increase in GAMMs correlates with tumor proliferation. However, it remains unclear why a higher density of GAMMs reflects a detrimental cellular composition. GAMMs (both peripheral macrophages and brain-intrinsic microglia) have been proposed as regulators of the transition from proneural to mesenchymal GBM subtype, mesenchymal being the signature with poor prognosis in this transition [31]. In our GBM cases, increased Iba-1 expression is seen in more proliferative tumors, which is consistent with the increased transcriptional levels of Iba-1 found in the mesenchymal GBM subtype [31].

As GAMMs are the major source of non-neoplastic cells, they are considered the major cellular component of the tumor stroma [7]. However, given that the location of the stroma versus tumor nest in GBM has not been clearly delineated, it is unclear how this may contribute to a negative outcome. It is generally assumed for other tumor types that the location of the microenvironment regulates tumor-associated macrophages function [15, 35], however, the role of the stromal phenotype of GAMMs in human GBM requires further analysis to be better defined.

Stroma and tumor nest are well mapped in many types of tumors [18], and fibroblasts appear to build stroma in breast cancer, gastric cancer, epithelial cancer and many others. Nevertheless, the cellular composition of stroma in brain tumors has scarcely been explored. Our data shows that GAMMs populate both microenvironments with increasing density in more proliferative tumors, however, according to our results, the difference in phenotype between stroma and tumor nest seems crucial. Our results indicate that the nature of the stromal areas can be distinguished by the expression of MHCII in human GBM. Interestingly, levels of different MHCII parameters increase with the Ki67 index, which conforms with the idea that the tumor-stroma ratio (TSR) is associated with poor prognosis, a low TSR being indicative of aggressiveness for some tumors [13, 22]. Our data here suggest that this also happens in GBM, showing low ratios in more proliferative tumors (calculation of GFAP/MHCII measured volume ratios shows: TSR for Ki67 30%, 3; Ki67 20%, 8; Ki67 10%, 33).

Increased numbers of GFAP^+^ cells, T cells, and GAMMs, correlated with increasing vascular network complexity, which implies an increase in neuroinflammation and tissue deterioration due to BTB disruption. Importantly, our 3D quantifications show accurate measurements expressed in precise volumetric units in contrast with previous estimations made solely in two dimensions (i.e., 6 µm-thick sections) [20].

Our data provide an in-depth analysis of GBM in thick sections, giving a more comprehensive understanding of the cellular distribution within human GBM. The technique enables 3D tissue imaging providing a novel visualization of the cellular heterogeneity of this aggressive and deadly tumor.

Herein we display only a small representative selection from a large repository of images that are available on request.

## Supplementary information

**Additional file 1: Supplementary Table S1, Table S2.** Neuropathological data of the GBM patients and antibodies used in the methodology.

**Additional file 2: Supplementary Figure S1.** Human samples selected for the study comply with bona fide GBM neuropathology. **a** Cellularity in normal CTX (N. CTX) compared with GBM, evidenced by thionine staining. **b** Presence of canonical hypoxic pseudopalisades in GBM biopsies. **c** Examples of aberrant mitoses. **d** Glomeruloid vessels. **e** Overexpression of GFAP and vimentin (VIM) evidenced by immunohistofluorescence and nuclear counterstaining (DAPI). **f** Gemistocytic formations in two representative GBM samples evidenced by GFAP and DAPI. **g** Quantification of cellularity using computer-generated “spots”. **h** Comparison of cellularity in normal CTX (N. CTX) and GBM (confocal image and 3D segmentation). **i** Representative images of increasing cellularity with Ki67 percentage provided by the clinical neuropathology. **j** Quantification of average cellularity by 3D stereological dissector in analyzed samples. **k** Correlation of the average cellularity per 3D dissector with the Ki67-group index.

**Additional file 3: Supplementary Figure S2.** Methodological Diagram (workflow). **a** Patients diagnosed with GBM underwent surgical resection and biopsies were prepared and preserved for thick sectioning. **b** Multi-color free-floating immunohistofluorescence was performed on thick sections. **c** High-resolution confocal images were captured on the samples considering x, y and z axes to generate 3D tissue blocks. **d** 3D systematic sampling of the sections was conducted covering GBM biopsies. **e** Images were processed by deconvolution defined by PSF. **f** Blend views and isosurfaces were conducted with specialized rendering software, to generate maps of measurable structures. **g** 3D rendering and visualization were performed considering the BV footprint. **h** Mosaics were built by computerized stitching of the tissue blocks systematic sampling. **i** Different vascular parameters were measured as well as the spatial relationships with other cellular structures or populations.

**Additional file 4: Supplementary Figure S3.** Identification of cortical location of GBM cells by GFAP marker. **a** Primate-specific GFAP^+^ interlaminar astrocytes extend long thin processes from cortical layer I to layer III or IV. **b** Varicose-projecting and interlaminar astrocytes mingle in layer III. **c** Protoplasmic astrocytes appear from layer II surrounding blood vessels. **d** Detail of a protoplasmic astrocyte. **e** Varicose-projecting astrocytes. **f** Detail of varicose projection. **g** Peri-tumoral reactive astrocytes in deeper layers. **h** Protoplasmic astrocyte in deeper layer. **i** and **j** Detail of peritumoral reactive astrocytes. **k** Polarized varicose astrocyte. **l** Tumoral massive GFAP expression. **m** Gemistocytic formations in tumor nest. **n** Diagram of distribution of GFAP-expressing cells in GBM-affected CTX.

**Additional file 5: Supplementary Figure S4.** Aberrant COL-IV deposition in GBM. **a** Concentric multilayered deposition (1), concentric layers and granulated dispersed COL-IV deposition (2), aberrant deposition in large vascular structures (3). **b** Multilayered and perforated glomeruloid vessel within a GFAP^+^ tumor nest region. **c** Mosaic of hypervascularized node in GBM, GFAP^+^ (red) neoplastic areas with high deposition of COL-IV (green); CD31^+^ endothelium (magenta) showing hypertrophic limits of a vascular barrier.

**Additional file 6: Supplementary Figure S5.** Vascular structure and location in pseudopalisades. **a** Diagram of the pseudopalisades (PP) structures surrounding necrotic focus (NF) and central blood vessel (BV). **b** Detail of the central BV and the hyper-cellular area forming the PP. **c** Rendering of two pseudopalisades with central NF. **d** Hypermosaic of a dense area of hypoxic pseudopalisades showing structural relationship of BVs with PPs stained by DAPI, GFAP, COL-IV and CD31. **e** MHCII macrophages are relegated to the vascular areas in the PPs.

**Additional file 7: Supplementary Video 1.** Protoplasmic astrocyte and blood vessels in normal CTX. 3D rotation and clipping plane of a GFAP+ astrocyte (red) in close proximity to blood vessels evidenced by COL-IV (green) and CD31 (magenta). Nuclei are stained with DAPI (blue).

**Additional file 8: Supplementary Video 2.** Blood vessel and glioma cells in GBM. 3D rotation and clipping plane of GFAP^+^ glioma cells (red) in close proximity to a large vessel evidenced by COL-IV (green) and CD31 (magenta). Nuclei are counterstained with DAPI (blue). 3D rendering from Patient 11.

**Additional file 9: Supplementary Video 3.** Blood vessel and glioma cells in GBM. 3D rotation and clipping plane of GFAP^+^ glioma cells surrounding a branched blood vessel immunostained for COL-IV (green) and CD31 (magenta). Nuclei are stained with DAPI (blue). 3D rendering from patient 3.1.

**Additional file 10: Supplementary Figure S6.** Glomeruloid vessels. **a** Three-dimensional reconstruction of a glomeruloid vessel in GBM patient to visualize GFAP^+^ cells (red), basement membrane (green), endothelium (magenta) and nuclei (blue). **b** Visualization of maximum projection and sections of the glomeruloid structure. **c** Distinct distribution of basement membrane and endothelium in glomeruloid vessels. **d** Relative fluorescence intensity profile of 4 channels from yellow line in **c**.

**Additional file 11: Supplementary Video 4.** Confocal planes through a 3D stack of a glomeruloid vessel from GBM. Endothelial cells are immunostained for CD31 (magenta), and basement membrane is labeled with COL-IV antibodies (green). Glioma cells are immunostained for GFAP and nuclei are evidenced by DAPI (blue). Both zenithal view (left) and rotated view (right) are displayed.

**Additional file 12: Supplementary Video 5.** 3D rotation of a GBM sample containing tumor nest and stroma. Isosurfaces of GFAP-rich (red) and MHCII-rich (magenta) areas are shown as well as COL-IV^+^ blood vessels.

## Data Availability

The datasets during and/or analysed during the current study are available from the corresponding author on reasonable request.

## Abbreviations

GFAP: Glial fibrillary acidic proteinCOL-IV
COL-IV: Collagen IV or Type-IV collagen
CD31: Cluster of differentiation 31
Iba-1: Ionized calcium binding adaptor molecule 1
MHCII: Major histocompatibility complex class II
CD3: Cluster of differentiation 3

## References

[1] Arvanitis CD, Ferraro GB, Jain RK (2020). The blood–brain barrier and blood–tumour barrier in brain tumours and metastases. Nat Rev Cancer.

[2] Barcia C, Gomez A, Gallego-Sanchez JM, Perez-Valles A, Castro MG, Lowenstein PR, Barcia C, Herrero MT (2009). Infiltrating CTLs in human glioblastoma establish immunological synapses with tumorigenic cells. Am J Pathol.

[3] Brat DJ, Castellano-Sanchez AA, Hunter SB, Pecot M, Cohen C, Hammond EH, Devi SN, Kaur B, Van Meir EG (2004). Pseudopalisades in glioblastoma are hypoxic, express extracellular matrix proteases, and are formed by an actively migrating cell population. Can Res.

[4] Das S, Marsden PA (2013). Angiogenesis in glioblastoma. N Engl J Med.

[5] Diaz LR, Saavedra-Lopez E, Romarate L, Mitxitorena I, Casanova PV, Cribaro GP, Gallego JM, Perez-Valles A, Forteza-Vila J, Alfaro-Cervello C, Garcia-Verdugo JM, Barcia C, Barcia C (2018). Imbalance of immunological synapse-kinapse states reflects tumor escape to immunity in glioblastoma. JCI Insight.

[6] Griveau A, Seano G, Shelton SJ, Kupp R, Jahangiri A, Obernier K, Krishnan S, Lindberg OR, Yuen TJ, Tien AC, Sabo JK, Wang N, Chen I, Kloepper J, Larrouquere L, Ghosh M, Tirosh I, Huillard E, Alvarez-Buylla A, Oldham MC, Persson AI, Weiss WA, Batchelor TT, Stemmer-Rachamimov A, Suva ML, Phillips JJ, Aghi MK, Mehta S, Jain RK, Rowitch DH (2018). A glial signature and Wnt7 signaling regulate glioma-vascular interactions and tumor microenvironment. Cancer Cell.

[7] Hambardzumyan D, Gutmann DH, Kettenmann H (2016). The role of microglia and macrophages in glioma maintenance and progression. Nat Neurosci.

[8] Hardee ME, Zagzag D (2012). Mechanisms of glioma-associated neovascularization. Am J Pathol.

[9] Jackson CM, Choi J, Lim M (2019). Mechanisms of immunotherapy resistance: lessons from glioblastoma. Nat Immunol.

[10] Jain RK, di Tomaso E, Duda DG, Loeffler JS, Sorensen AG, Batchelor TT (2007). Angiogenesis in brain tumours. Nat Rev Neurosci.

[11] Jonkman J, Brown CM, Wright GD, Anderson KI, North AJ (2020). Tutorial: guidance for quantitative confocal microscopy. Nat Protoc.

[12] Kreuger J, Phillipson M (2016). Targeting vascular and leukocyte communication in angiogenesis, inflammation and fibrosis. Nat Rev Drug Discov.

[13] Lee D, Ham IH, Son SY, Han SU, Kim YB, Hur H (2017). Intratumor stromal proportion predicts aggressive phenotype of gastric signet ring cell carcinomas. Gastric Cancer: Off J Int Gastric Cancer Assoc Jpn Gastric Cancer Assoc.

[14] Lee JH, Lee JE, Kahng JY, Kim SH, Park JS, Yoon SJ, Um JY, Kim WK, Lee JK, Park J, Kim EH, Lee JH, Lee JH, Chung WS, Ju YS, Park SH, Chang JH, Kang SG, Lee JH (2018). Human glioblastoma arises from subventricular zone cells with low-level driver mutations. Nature.

[15] Lewis CE, Pollard JW (2006). Distinct role of macrophages in different tumor microenvironments. Can Res.

[16] Lim M, Xia Y, Bettegowda C, Weller M (2018). Current state of immunotherapy for glioblastoma. Nat Rev Clin Oncol.

[17] McGeer PL, Itagaki S, McGeer EG (1988). Expression of the histocompatibility glycoprotein HLA-DR in neurological disease. Acta Neuropathol.

[18] Mueller MM, Fusenig NE (2004). Friends or foes—bipolar effects of the tumour stroma in cancer. Nat Rev Cancer.

[19] Munn LL, Jain RK (2019). Vascular regulation of antitumor immunity. Science.

[20] Nishie A, Ono M, Shono T, Fukushi J, Otsubo M, Onoue H, Ito Y, Inamura T, Ikezaki K, Fukui M, Iwaki T, Kuwano M (1999). Macrophage infiltration and heme oxygenase-1 expression correlate with angiogenesis in human gliomas. Clin Cancer Res: Off J Am Assoc Cancer Res.

[21] Nyengaard JR, Bendtsen TF, Gundersen HJ (1988). Stereological estimation of the number of capillaries, exemplified by renal glomeruli. APMIS Suppl.

[22] Peng C, Liu J, Yang G, Li Y (2018). The tumor-stromal ratio as a strong prognosticator for advanced gastric cancer patients: proposal of a new TSNM staging system. J Gastroenterol.

[23] Piali L, Hammel P, Uherek C, Bachmann F, Gisler RH, Dunon D, Imhof BA (1995). CD31/PECAM-1 is a ligand for alpha v beta 3 integrin involved in adhesion of leukocytes to endothelium. J Cell Biol.

[24] Poon CC, Sarkar S, Yong VW, Kelly JJP (2017). Glioblastoma-associated microglia and macrophages: targets for therapies to improve prognosis. Brain: J Neurol.

[25] Pries AR, Cornelissen AJ, Sloot AA, Hinkeldey M, Dreher MR, Hopfner M, Dewhirst MW, Secomb TW (2009). Structural adaptation and heterogeneity of normal and tumor microvascular networks. PLoS Comput Biol.

[26] Quail DF, Joyce JA (2017). The microenvironmental landscape of brain tumors. Cancer Cell.

[27] Richter M, Ray SJ, Chapman TJ, Austin SJ, Rebhahn J, Mosmann TR, Gardner H, Kotelianski V, deFougerolles AR, Topham DJ (2007). Collagen distribution and expression of collagen-binding alpha1beta1 (VLA-1) and alpha2beta1 (VLA-2) integrins on CD4 and CD8 T cells during influenza infection. J Immunol.

[28] Roberts AI, Brolin RE, Ebert EC (1999). Integrin alpha1beta1 (VLA-1) mediates adhesion of activated intraepithelial lymphocytes to collagen. Immunology.

[29] Saavedra-López E, Roig-Martínez M, Cribaro GP, Casanova PV, Gallego JM, Pérez-Vallés A, Barcia C (2019). Phagocytic glioblastoma-associated microglia and macrophages populate invading pseudopalisades. Brain Commun.

[30] Sindhwani S, Syed AM, Ngai J, Kingston BR, Maiorino L, Rothschild J, MacMillan P, Zhang Y, Rajesh NU, Hoang T, Wu JLY, Wilhelm S, Zilman A, Gadde S, Sulaiman A, Ouyang B, Lin Z, Wang L, Egeblad M, Chan WCW (2020). The entry of nanoparticles into solid tumours. Nat Mater.

[31] Wang Q, Hu B, Hu X, Kim H, Squatrito M, Scarpace L, deCarvalho AC, Lyu S, Li P, Li Y, Barthel F, Cho HJ, Lin YH, Satani N, Martinez-Ledesma E, Zheng S, Chang E, Sauve CG, Olar A, Lan ZD, Finocchiaro G, Phillips JJ, Berger MS, Gabrusiewicz KR, Wang G, Eskilsson E, Hu J, Mikkelsen T, DePinho RA, Muller F, Heimberger AB, Sulman EP, Nam DH, Verhaak RGW (2017). Tumor evolution of glioma-intrinsic gene expression subtypes associates with immunological changes in the microenvironment. Cancer Cell.

[32] Watkins S, Robel S, Kimbrough IF, Robert SM, Ellis-Davies G, Sontheimer H (2014). Disruption of astrocyte-vascular coupling and the blood-brain barrier by invading glioma cells. Nat Commun.

[33] Winkler F, Kienast Y, Fuhrmann M, Von Baumgarten L, Burgold S, Mitteregger G, Kretzschmar H, Herms J (2009). Imaging glioma cell invasion in vivo reveals mechanisms of dissemination and peritumoral angiogenesis. Glia.

[34] Wolf KJ, Chen J, Coombes JD, Aghi MK, Kumar S (2019). Dissecting and rebuilding the glioblastoma microenvironment with engineered materials. Nat Rev Mater.

[35] Yang M, McKay D, Pollard JW, Lewis CE (2018). Diverse functions of macrophages in different tumor microenvironments. Can Res.

