# Supplementary material for: Three-dimensional vascular microenvironment landscape in human glioblastoma
Source: Acta Neuropathol Commun. 2021 Feb 12;9:24. doi: 10.1186/s40478-020-01115-0 (PMC7879533; doi:10.1186/s40478-020-01115-0)
Supplement: Supplementary file 1 — Additional file 1: Supplementary Table S1, Table S2. Neuropathological data of the GBM patients and antibodies used in the methodology. [file 40478_2020_1115_MOESM1_ESM.docx]

**Supplementary table S1**

| **Sample no.** | **Lab reference no.** | **Glioma grade** | **Ki67 index (%)** | **Vimentin** | **GV** | **PP** | **Aberrant Mitoses** | **Location** |
| --- | --- | --- | --- | --- | --- | --- | --- | --- |
| 1 | 11 | 4 | 10 | + | + | NR | + | right temporal - frontal |
| 2 | 2 |  | 20 | NR | ++ | NR | + | right temporal - frontal |
| 3 | 3.2 |  |  | ++ | + | NR | + | left frontal |
| 4 | 3.4 |  |  | +++ | ++ | +++ | + | left parietal |
| 5 | 3.1 |  | 30 | ++ | +++ | ++ | +++ | left temporal |
| 6 | 3.6 |  |  | +++ | + | + | ++ | left temporal |
| 7 | 4 |  |  | NR | ++ | ++ | ++ | insular |
| 8 | 12 |  | 50 | ++++ | + | NR | + | right temporal - frontal |

*NR: Not reported on medical record

| **Supplementary table S2.** | | |  |  |  | |
| --- | --- | --- | --- | --- | --- | --- |
|  |  | |  |  |  | |
| **Primary antibodies** |  | |  |  |  | |
| **Antigen** | **Company** | | **Catalog number** | **Species Reactivity** | **Host Species** |  |
| Collagen IV | Abcam, Cambridge, UK | | ab6586 | Mouse, rat, hamster, cow, dog, human, pig, zebrafish, African green monkey, Chinese hamster, Syrian hamster | polyclonal rabbit (IgG) |  |
| GFAP | Millipore, Temecula, CA, USA | | AB5804 | African green monkey, Trachemys dorbigni, human, mouse, rat, dog, cow, Rhesus monkey | polyclonal rabbit |  |
| GFAP | Abcam, Cambridge, UK | | ab4674 | Mouse, rat, rabbit, chicken, cow, human, Rhesus monkey, Apteronotus leptorhynchus | polyclonal chicken (IgY) |  |
| CD31 | Abcam, Cambridge, UK | | ab199012 | Human, Cynomolgus monkey | monoclonal mouse (IgG1) |  |
| HLA-DP, DQ, DR | Dako Cytomation; Glostrup, Denmark | | M0775 | Human | monoclonal mouse (IgG1) |  |
| CD3 | Dako Cytomation; Glostrup, Denmark | | A0452 | Tasmanian devil, African green monkey, crab-eating macaque, human, mouse, rat, dog, sheep, equine, Rhesus monkey | polyclonal rabbit |  |
| Iba-1 | Wako Pure Chemical Industries, Ltd.; Osaka, Japan | | 019-19741 | Styela clava, domestic ferret, African green monkey, hamsters, human, mouse, rat, dog, pig, sheep, Rhesus monkey | polyconal rabbit (IgG) |  |
|  |  | |  |  |  |  |
|  |  | |  |  |  |  |
| **Secondary antibodies** | | |  |  |  |  |
| **Conjugate** | | **Company** | **Catalog number** | **Reactivity** | **Species*** |  |
| Alexa Fluor 488 | | Invitrogen, Carlsbad, CA, USA | A-11008 | rabbit | goat (IgG) |  |
| Alexa Fluor 555 | | Invitrogen, Carlsbad, CA, USA | A-21422 | mouse | goat (IgG) |  |
| Alexa Fluor 555 | | Invitrogen, Carlsbad, CA, USA | A-21437 | chicken | goat (IgG) |  |
| Alexa Fluor 647 | | Invitrogen, Carlsbad, CA, USA | A-21235 | mouse | goat (IgG) |  |

**Glossary of markers**

**GFAP (Glial fibrillary acidic protein):** A member of the cytoskeletal protein family widely expressed in astroglial cells and in tumor cells with astrocytic phenotype, such as astrocytoma and thus GBM. A marker widely used and accepted for GBM diagnostics.

**COL-IV (Collagen IV or Type-IV collagen):** Integral component of blood vessel basement membranes and a reliable marker for blood vessels in tissue.

**CD31 (Cluster of differentiation 31):** membrane protein expressed on the surface of endothelial cells, also known as platelet endothelial cell adhesion molecule-1 (PECAM-1), which is generally used to detect vascular endothelium and are known to proliferate in GBM.

**Iba-1 (Ionized calcium binding adaptor molecule 1):** Microglial and macrophage-specific calcium-binding protein that is a reliable marker to detect this cell population.

**MHCII (major histocompatibility complex class II):** Specifically, the human form HLA-DR (Human Leukocyte Antigen DR) is a marker normally used for microglia and macrophage activation, although other antigen presenting cells such as dendritic cells or mononuclear phagocytes can also express it.

**CD3 (Cluster of differentiation 3):** A membrane-bound antigen found in all mature T cells and acting as a T-cell co-receptor that helps activate cytotoxic T cells and T helper cells.
