# Supplementary figures and images for: Three-dimensional vascular microenvironment landscape in human glioblastoma
Source: Acta Neuropathol Commun. 2021 Feb 12;9:24. doi: 10.1186/s40478-020-01115-0 (PMC7879533; doi:10.1186/s40478-020-01115-0)

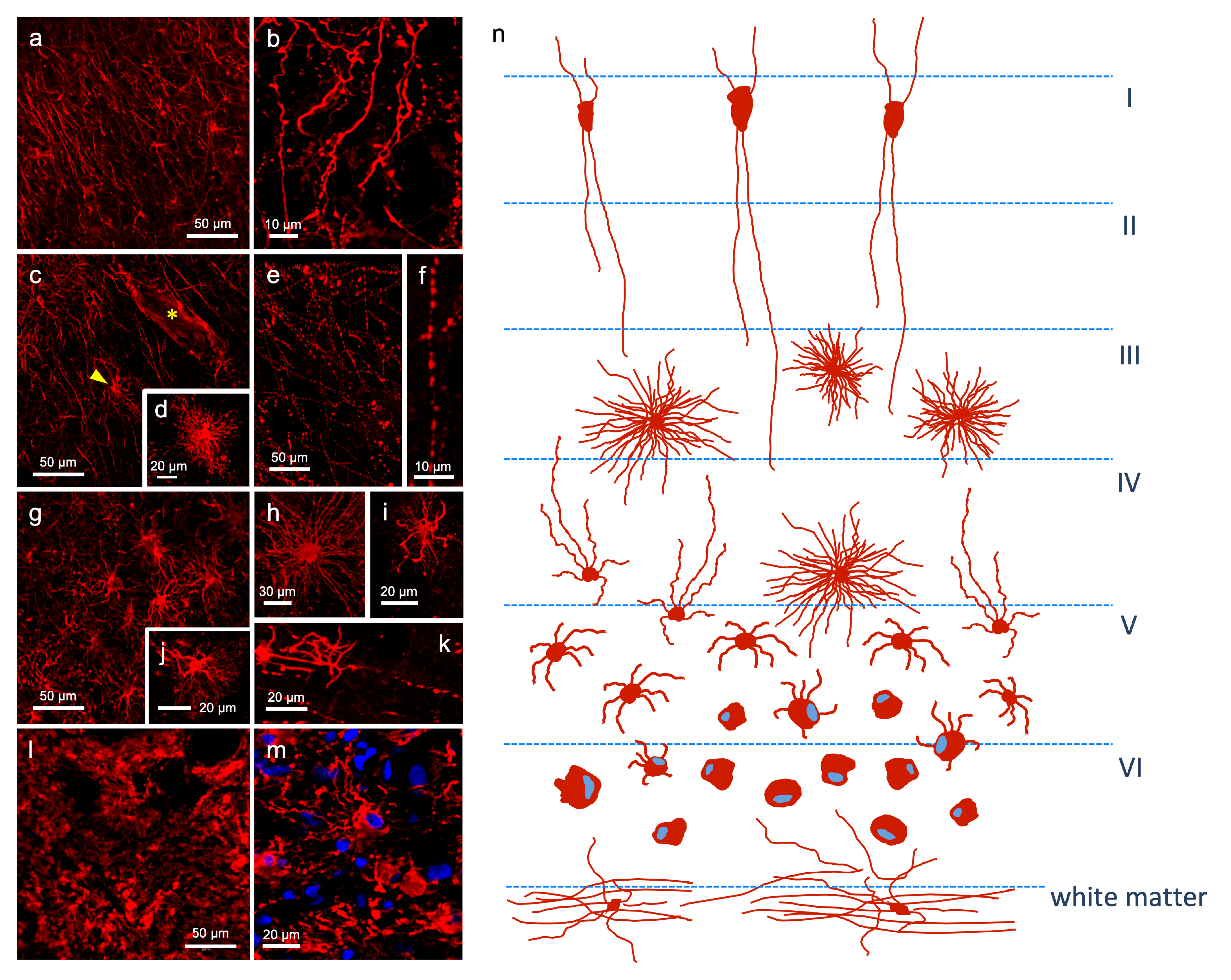

Supplement: Supplementary file 4 — Additional file 4: Supplementary Figure S3. Identification of cortical location of GBM cells by GFAP marker. a Primate-specific GFAP+ interlaminar astrocytes extend long thin processes from cortical layer I to layer III or IV. b Varicose-projecting and interlaminar astrocytes mingle in layer III. c Protoplasmic astrocytes appear from layer II surrounding blood vessels. d Detail of a protoplasmic astrocyte. e Varicose-projecting astrocytes. f Detail of varicose projection. g Peri-tumoral reactive astrocytes in deeper layers. h Protoplasmic astrocyte in deeper layer. i and j Detail of peritumoral reactive astrocytes. k Polarized varicose astrocyte. l Tumoral massive GFAP expression. m Gemistocytic formations in tumor nest. n Diagram of distribution of GFAP-expressing cells in GBM-affected CTX. [file 40478_2020_1115_MOESM4_ESM.tiff]

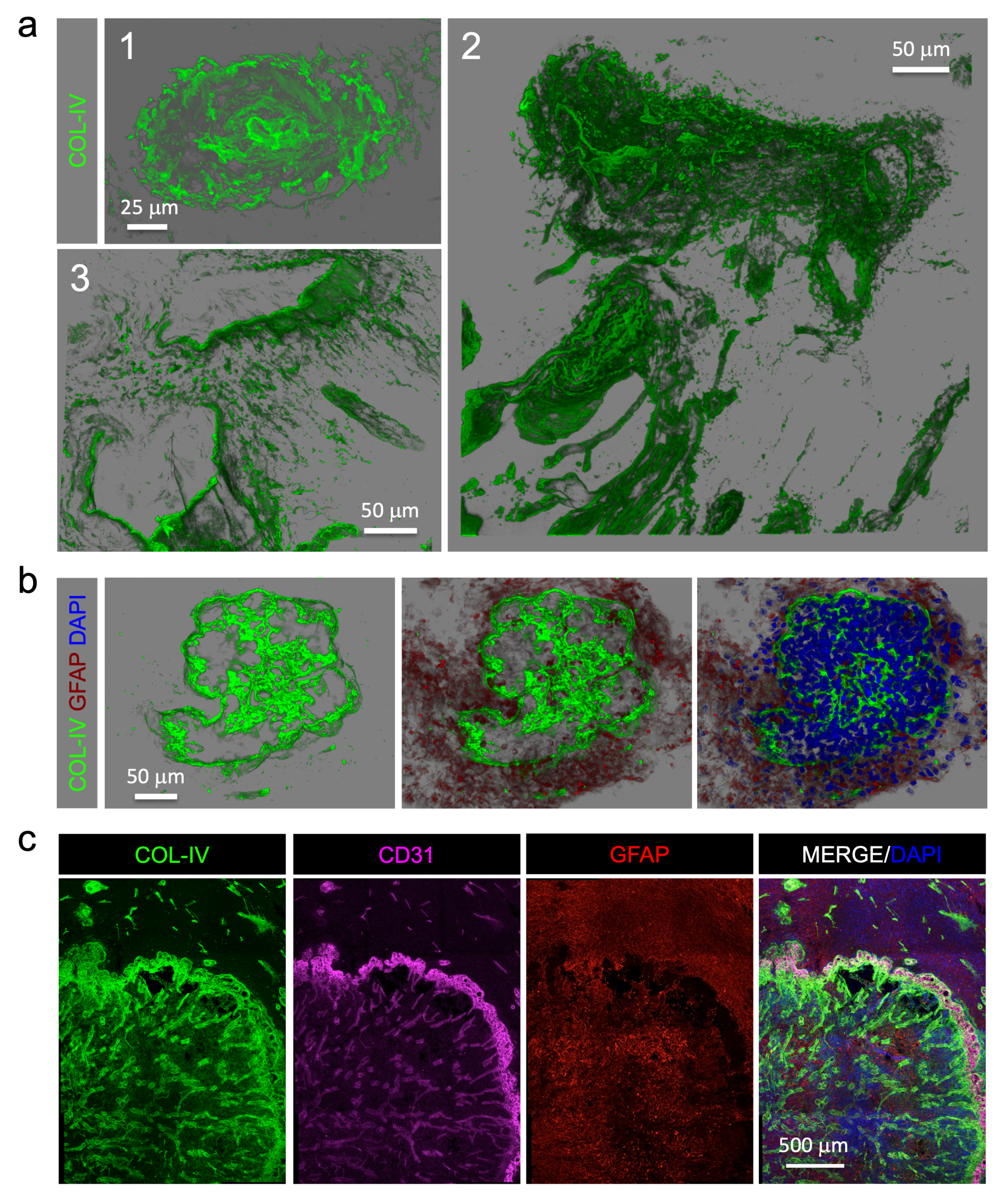

Supplement: Supplementary file 5 — Additional file 5: Supplementary Figure S4. Aberrant COL-IV deposition in GBM. a Concentric multilayered deposition (1), concentric layers and granulated dispersed COL-IV deposition (2), aberrant deposition in large vascular structures (3). b Multilayered and perforated glomeruloid vessel within a GFAP+ tumor nest region. c Mosaic of hypervascularized node in GBM, GFAP+ (red) neoplastic areas with high deposition of COL-IV (green); CD31+ endothelium (magenta) showing hypertrophic limits of a vascular barrier. [file 40478_2020_1115_MOESM5_ESM.tiff]

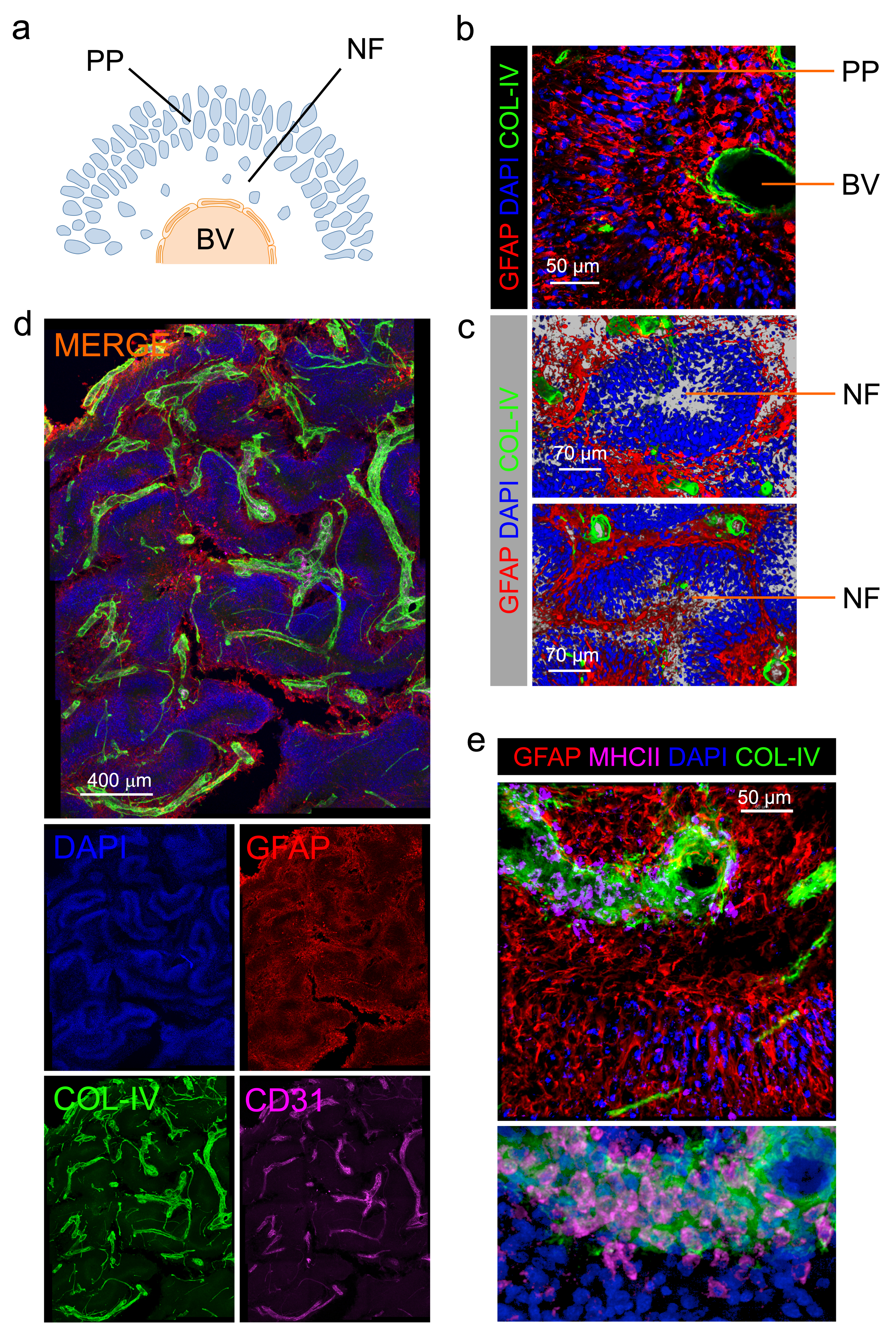

Supplement: Supplementary file 6 — Additional file 6: Supplementary Figure S5. Vascular structure and location in pseudopalisades. a Diagram of the pseudopalisades (PP) structures surrounding necrotic focus (NF) and central blood vessel (BV). b Detail of the central BV and the hyper-cellular area forming the PP. c Rendering of two pseudopalisades with central NF. d Hypermosaic of a dense area of hypoxic pseudopalisades showing structural relationship of BVs with PPs stained by DAPI, GFAP, COL-IV and CD31. e MHCII macrophages are relegated to the vascular areas in the PPs. [file 40478_2020_1115_MOESM6_ESM.tiff]
